# Associations between mid‐life social relationships and the risk of incident dementia: The ARIC study

**DOI:** 10.1002/alz.70365

**Published:** 2025-07-14

**Authors:** Renée C. Groechel, Albert C. Liu, Pamela L. Lutsey, Priya Palta, Anna M. Kucharska‐Newton, Silvia Koton, A. Richey Sharrett, Alden L. Gross, Keenan A. Walker, Rebecca F. Gottesman

**Affiliations:** ^1^ National Institute of Neurological Disorders & Stroke Intramural Research Program, National Institutes of Health Bethesda Maryland USA; ^2^ Department of Epidemiology University of North Carolina Gillings School of Global Public Health Chapel Hill North Carolina USA; ^3^ Division of Epidemiology and Community Health University of Minnesota School of Public Health Minneapolis Minnesota USA; ^4^ Department of Neurology University of North Carolina at Chapel Hill Chapel Hill North Carolina USA; ^5^ Department of Nursing The Stanley Steyer School of Health Professions, Tel Aviv University Tel Aviv Israel; ^6^ Department of Epidemiology Johns Hopkins University Bloomberg School of Public Health Baltimore Maryland USA; ^7^ National Institute on Aging Intramural Research Program, National Institutes of Health Baltimore Maryland USA

**Keywords:** Atherosclerosis Risk in Communities (ARIC) study, dementia, longitudinal follow‐up, mid‐life, psychosocial health, social relationships

## Abstract

**INTRODUCTION:**

This study aimed to assess whether mid‐life social relationships are associated with a lower risk of late‐life dementia.

**METHODS:**

Participants in the Atherosclerosis Risk in Communities (ARIC) study were assessed for social support and isolation (Visit 2;1990–1992). A composite measure, “social relationships,” was generated. Incident dementia cases were identified following Visit 2 through 2019, using ongoing surveillance. Associations between mid‐life social relationships and incident dementia were evaluated with Cox proportional‐hazard regression models. Formal interaction tests examined whether sex or race modified this association.

**RESULTS:**

Among 13,070 participants without dementia, those with strong social relationships in mid‐life had a lower risk for developing dementia, compared to participants with poor mid‐life social relationships. Neither sex nor race significantly modified this association.

**DISCUSSION:**

Stronger mid‐life social relationships may have a potentially protective effect on dementia risk. Future studies evaluating psychosocial health at multiple time points in ethnically diverse populations are needed.

**Highlights:**

- Psychosocial health is a modifiable risk factor for dementia.
- Stronger mid‐life social relationships are associated with a lower risk of dementia.
- Social relationships may have a potentially protective effect on late‐life outcomes.
- This study leveraged data collected from nearly 20 years of follow‐up in 13,070 participants.
- Longitudinal data used in this study were captured in a population that is 24% Black.

## 1 BACKGROUND

Psychosocial health has gained widespread recognition as a key factor in preserving cognition and sustained functional independence in aging adults.^1^, ^2^ As such, numerous observational cohort studies have examined the association between different indices of psychosocial health and related social behaviors with long‐term cognitive function and the risk of incident dementia.^3^, ^4^

Many of these studies examining the association between psychosocial health with dementia and related late‐life outcomes remain limited to small (*n* <210) ^5^, ^6^, ^7^ or racially homogeneous samples,^8^, ^9^, ^10^, ^11^, ^12^ making findings less generalizable to the broader population. Others are cross‐sectional^13^, ^14^ or have follow‐up periods of less than 10 years, rendering them susceptible to reverse causation.^6^, ^7^, ^9^, ^15^, ^16^, ^17^, ^18^ Still, other studies have evaluated the impact of psychosocial health over longer follow‐up periods, but the outcome of interest is cognitive decline in specified domains (e.g., processing speed, verbal memory, executive function).^7^, ^14^, ^19^, ^20^ Altogether, findings from these studies have indicated that psychosocial health may have a potentially protective effect on late‐life outcomes,^4^, ^5^, ^7^, ^10^, ^11^, ^13^, ^21^ although the reported magnitude of this association varies likely due to differences in study design, the constructs used to define psychosocial health, and the outcomes examined.

Through data from 13,070 U.S. adults enrolled in the community‐based Atherosclerosis Risk in Communities (ARIC) cohort, we hypothesized that participants with stronger social relationships in mid‐life would have a lower risk of dementia in late‐life. The component of psychosocial health assessed was “social relationships,” operationalized through joint classification of measures used to categorize perceived social support and social isolation. Two factors were key to our investigation: (1) the use of a follow‐up period that was nearly 20 years long, which allowed us to more accurately report observed associations between social relationships and dementia risk; and (2) the examination of incident dementia as the outcome, due to its growing prevalence underscored by increasing necessity to understand how components of psychosocial health relate to clinical change substantial enough to warrant a medical diagnosis.

Secondary analyses examined effect modification of the association between social relationships in mid‐life and dementia risk in late‐life by sex and race. Finally, due to growing interest in understanding the interplay between co‐existing genetic factors and additional lifestyle factors adjacent to psychosocial health,^22^ we further examined whether these factors modify the association between social relationships in mid‐life and dementia risk.

## 2 METHODS

### 2.1 Study population

**FIGURE 1 alz70365-fig-0001:**
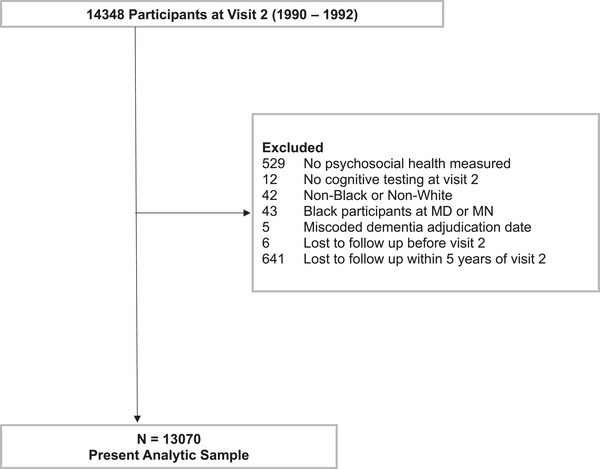
Exclusion criteria for analytic sample (*N* = 13,070). Lost to follow‐up including but not limited to participants who developed dementia and/or died.

The ARIC study recruited 15,792 adults (aged 45 to 64 years) from four U.S. communities at baseline (Visit 1:1987–1989).^23^ Self‐reported questionnaires regarding psychosocial measures were collected in 14,348 participants at Visit 2 (1990–1992). Cognition was further assessed at Visit 2 using a three‐instrument cognitive assessment. Only participants who did not have dementia and had psychosocial health assessed at Visit 2 were included in the present study. Follow‐up for development of incident dementia was though December 31, 2019. For roughly 30% of participants seen at the Jackson site, follow‐up data for this study was censored through December 31, 2017, due to administrative delays in data reporting. Further details of the study timeline (Figure S1) and exclusion criteria are described (Figure 1). In total, the study used longitudinal data from 13,070 participants. The study was approved by institutional review boards at each study center, and all participants provided written informed consent to participate in each study visit and follow‐up. 

### 2.2 Psychosocial measures

Perceived social support was evaluated at Visit 2 using the Interpersonal Support Evaluation List‐Short Form (ISEL‐SF; Figure S2). This 16‐item scale was constructed and validated by ARIC investigators from the original 40‐item full scale and assesses perceived social support with four subscales including (1) appraisal support, (2) tangible assets support, (3) belonging support, and (4) self‐esteem support.^24^, ^25^ The total score is an equally weighted sum, with a range of 0–48. Higher scores indicate greater perceived social support. Total ISEL‐SF score was categorized into distribution‐based tertiles of the full ARIC sample assessed at Visit 2 (high ≥42, intermediate 36–41, and low ≤35).^26^

Social isolation was evaluated using the Lubben Social Network Scale (LSNS; Figure S3), also administered at Visit 2. This 10‐item scale assesses the size of the participant's active social network and the perceived social support received by family, friends, and neighbors. The total score is an equally weighted sum, with a range of 0–50. Higher scores indicate a lower risk of social isolation. Scores were divided into four categories based upon the Lubben criteria, which has been used in many analyses conducted in the ARIC cohort: ≤20 = isolated; 21–25 = high risk for isolation; 26–30 = moderate risk for isolation; ≥31 = low risk for isolation.^19^, ^26^, ^27^, ^28^

**FIGURE 2 alz70365-fig-0002:**
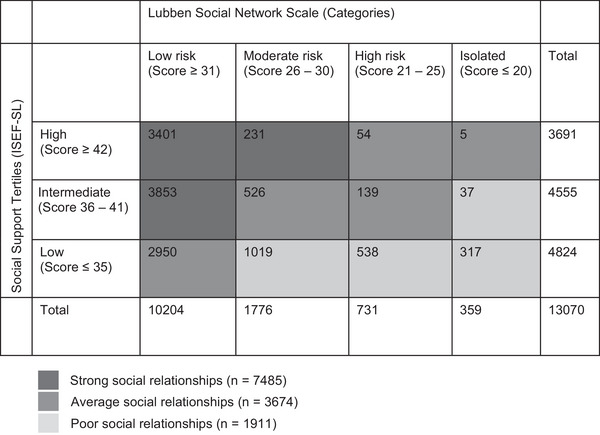
Categorization of social relationships (*N* = 13,070).

Following the categorization of both psychosocial measures, participants fell into 12 different groups. Performance on the ISEL‐SF and LSNS was paired to create a measure reflecting social relationships (Figure 2). Participants were classified as having strong, average, or poor social relationships in mid‐life. This conversion into a categorical composite measure that encompasses both psychosocial measures was conducted for several reasons. First, the correlation between continuous ISEL‐SF and LSNS scores in this sample is moderate (*r* = 0.46), which may indicate that the two constructs are not entirely linear with one another. Second, previous studies in the ARIC‐PET (positron emission tomography) cohort have shown stronger associations with dementia risk when using the “social relationships” variable as opposed to independent scales.^29^ Finally, the use of an integrative, composite measure allows us to assess multiple facets of psychosocial health more comprehensively, which aligns with recent recommendations to study healthy aging with a holistic approach.^21^, ^22^, ^30^, ^31^

### 2.3 Dementia

The 3‐instrument cognitive battery (Delayed Word Recall, Word Fluency, and Digit Symbol Substitution) initially used at Visit 2 was repeated at Visit 4 (1996–1998), and an expanded battery was administered at Visit 5 as part of the ARIC Neurocognitive Study (ARIC‐NCS; 2011–2013).^32^, ^33^, ^34^ Incident dementia cases were identified in several ways. First, as part of the ARIC‐NCS, participants seen in‐person for Visits 5–7 underwent detailed cognitive testing, and a subset had informant interviews. Participants seen in‐person for Visits 5–7 underwent a physician‐ and neuropsychologist‐led adjudication process and standard diagnostic criteria, which led to a research diagnosis of normal cognition, mild cognitive impairment (MCI), or dementia.^32^, ^33^, ^34^ Second, in addition to in‐person visits, dementia surveillance was ongoing by use of telephone‐based cognitive assessments, informant interviews, and hospitalization codes and death certificates. Additional post‐Visit 5 incident dementia cases were identified through several sources, including administration of the Six Item Screener (SIS) annually by phone and the Ascertain Dementia 8‐Item Informant Questionnaire (AD8), when appropriate.^32^, ^33^, ^34^ Date of dementia onset was defined as the earliest date of SIS/AD8 interviews, date of hospitalization records with dementia diagnosis, or the date of the in‐person visit when a participant was classified as having dementia. Of participants with diagnoses based on SIS/AD8, hospitalization records, or death certificates, dementia onset was defined as 180 days prior to the interview, hospitalization, or death.^34^, ^35^, ^36^

**RESEARCH IN CONTEXT**

1. **Systematic review**: The authors reviewed the literature using PubMed for studies examining psychosocial health and long‐term health outcomes. This is a growing area of interest, yet there remains a strong need for longitudinal studies that specifically assess the association between behaviors underlying social relationships with dementia as the primary outcome. Relevant existing publications are cited.
2. **Interpretation**: In this large community‐based cohort of 13,070 individuals with roughly 20 years of follow‐up, our results provide important evidence that stronger social relationships in mid‐life are associated with a lower risk of incident dementia in late‐life.
3. **Future directions**: Our findings provide further evidence of the potentially protective role of strong psychosocial health in mid‐life. Future studies can build upon these findings by continuing to evaluate psychosocial health throughout the life course in racial and ethnically diverse populations with additional characterization of related lifestyle factors and social determinants of health.

### 2.4 Covariates

Covariates included age (at Visit 2), sex, educational attainment (less than high school, high school or equivalent, or more than high school), race, and apolipoprotein E (*APOE*) ɛ4 genotype (0 or ≥1 allele). Race and study center were combined[Fig alz70365-fig-0001] to[Fig alz70365-fig-0002] create a race‐center variable that accounted for the distribution of racial groups across ARIC centers. Occupational and marital status were self‐reported at Visit 2 and categorized as “employed and working outside the home” or “not working outside the home” (which included being retired, unemployed, or a homemaker) and “married” or “not married” (divorced, separated, widowed, or never married), respectively. The Maastricht Vital Exhaustion Questionnaire, administered at Visit 2 to measure symptoms of depression and fatigue, was dichotomized at ≥14 to indicate depressive symptomology_._ ^37^, ^38^, ^39^ Vascular risk factors, including hypertension (systolic blood pressure >140 mm Hg, diastolic blood pressure >90 mm Hg, or use of antihypertensive medications), diabetes (fasting glucose ≥126 mg/dL, non‐fasting glucose ≥200 mg/dL, HbA1c ≥6.5, self‐report of physician‐diagnosed diabetes, or use of oral diabetes medications or insulin), smoking/drinking (self‐report, dichotomized into ever vs never), obesity (BMI ≥30 kg/m^2^), and elevated total cholesterol (≥200 mg/dL) were collected at Visit 2.

### 2.5 Statistical analysis

R version 4.3.1 (R Foundation for Statistical Computing) was used for all analyses. Participant characteristics were evaluated, and Cox proportional‐hazards regression models were used to estimate hazard ratios (HRs) and 95% confidence intervals (CIs) for the association between social relationships and incident dementia from ARIC Visit 2 through December 31, 2019 (with 5 years after Visit 2 considered to be time 0). Three covariate‐adjusted models were fit to the data: Model 1 adjusted for demographics and *APOE* ɛ4; Model 2 additionally adjusted for occupational status, marital status, and depressive symptoms at Visit 2; and Model 3 further adjusted for vascular risk factors measured at Visit 2. Because of the possibility that vascular risk factors are on the causal pathway between psychosocial measures and dementia (and thus are not potential confounders), our primary analyses and inferences were based on Model 2 because Model 3 may be over‐adjusted. Secondary analyses explored the association between social relationships and incident dementia with Wald tests for interaction by sex and race, prior to stratification by both measures. In addition, we evaluated interactions between social relationships with age (dichotomized at the Visit 2 sample median), *APOE* ɛ4 genotype, depressive symptoms, and educational attainment. *P* < 0.05 was considered statistically significant; testing was two‐sided. Multiple imputation chained equations (MICE) were used to impute missing covariates in 820 participants.

### 2.6 Sensitivity analyses

Sensitivity analyses assessed the independent contributions of social support and social isolation as categorical measures, since these factors were paired to create the “social relationships” variable. The purpose of these analyses was to see whether the observed effects of psychosocial measures on dementia risk were congruent across these different behaviors comprising the social relationships variable. Additional sensitivity analyses were conducted, excluding participants whose global cognitive function fell in the lowest fifth percentile at Visit 2 or anyone who had an incident stroke prior to the end of follow‐up. These analyses were conducted to confirm that the associations shown were not driven by those at the highest risk of dementia due to extrinsic factors such as low baseline cognitive function or stroke. We performed a Fine and Gray sub‐distribution competing risk analysis with death as a competing outcome.^40^

## 3 RESULTS

### 3.1 Participant characteristics

The analytic sample included 13,070 participants (7268 [56%] women, 3127 [24%] Black, Visit 2 median [interquartile range; IQR] age, 57 [10] years). Fifty‐seven percent of participants had “high” or “intermediate” social support and risk of social isolation was “low” or “moderate,” and they were classified as having strong mid‐life social relationships, whereas 28% had average mid‐life social relationships, and 15% had poor mid‐life social relationships (Figure 2). The median (IQR) follow‐up (after accounting for a 5‐year lag exposure period following Visit 2) was 19.3 (9.1) years, during which 2643 (20%) of participants developed dementia (Table 1). The incidence rate (per 1000 person‐years) was 14.2 for participants with poor mid‐life social relationships, 12.7 for those with average mid‐life social relationships, and 11.1 for those with strong mid‐life social relationships.

**TABLE 1:** ARIC participant characteristics of the analytic sample at Visit 2 (1990–1992); stratified by level of social relationships (*N* = 13,070).

Characteristics	Full sample (*N* = 13,070)	Strong social relationships (*n* = 7485)	Average social relationships (*n* = 3674)	Poor social relationships (*n* = 1911)	*p‐*value
Age, y, median (Q1, Q3)	57 (52, 62)	56 (52, 61)	57 (52, 62)	57 (52, 62)	0.001
Sex, *n* (%)					<0.001
Women	7268 (55.6)	4417 (59.0)	1954 (53.2)	897 (46.9)	
Men	5802 (44.4)	3068 (41.0)	1720 (46.8)	1014 (53.1)	
Race‐ARIC Center, *n* (%)					0.003
Black – Forsyth	353 (2.7)	191 (2.6)	93 (2.5)	69 (3.6)	
Black – Jackson	2774 (21.2)	1514 (20.2)	820 (22.3)	440 (23.0)	
White – Forsyth	3119 (23.9)	1863 (24.9)	853 (23.2)	405 (21.2)	
White – Minnesota	3534 (27.0)	2056 (27.5)	992 (27.0)	486 (25.4)	
White – Washington	3290 (25.2)	1861 (24.9)	916 (24.9)	511 (26.7)	
*APOE* ɛ4 status, *n* (%)					0.04
Non‐carrier	8763 (67.0)	5100 (68.1)	2413 (65.7)	1250 (65.4)	
Carrier	3894 (29.8)	2161 (28.9)	1145 (31.2)	588 (30.8)	
Education level, *n* (%)					<0.001
Less than high school	2682 (20.5)	1297 (17.3)	894 (24.3)	491 (25.7)	
High school or equivalent	4101 (31.4)	2352 (31.4)	1173 (31.9)	576 (30.1)	
More than high school	6269 (48.0)	3828 (51.1)	1600 (43.5)	841 (44.0)	
Occupational status, *n* (%)					0.22
Employed outside the home	8694 (66.5)	5032 (67.2)	2420 (65.9)	1242 (65.0)	
Not working outside the home^a^	4376 (33.5)	2453 (32.8)	1254 (34.1)	669 (35.0)	
Marital status, *n* (%)					<0.001
Married	10,399 (79.6)	6275 (83.8)	2917 (79.4)	1207 (63.2)	
Not married^b^	2658 (20.3)	1202 (16.1)	753 (20.5)	703 (36.8)	
Depressive symptoms,^c^ *n* (%)	3847 (29.4)	1528 (20.4)	1400 (38.1)	919 (48.1)	<0.001
Hypertension, *n* (%)	4557 (34.9)	2511 (33.5)	1334 (36.3)	712 (37.3)	0.004
Diabetes, *n* (%)	1840 (14.1)	983 (13.1)	561 (15.3)	296 (15.5)	0.004
Body mass index (≥30 kg/m^2^), *n* (%)	3758 (28.8)	2113 (28.2)	1106 (30.1)	539 (28.2)	0.21
Total cholesterol (≥200 mg/dL), *n* (%)	7386 (56.5)	4233 (56.6)	2094 (57.0)	1059 (55.4)	0.70
Ever smoker, *n* (%)	7808 (59.7)	4238 (56.6)	2302 (62.7)	1268 (66.4)	<0.001
Ever drinker, *n* (%)	10,153 (77.7)	5719 (76.4)	2862 (77.9)	1572 (82.3)	<0.001
Developed dementia by Visit 7, *n* (%)	2643 (20.2)	1449 (19.4)	770 (21.0)	424 (22.2)	0.03

*Notes*: Data for the following covariates were missing and are not included in the proportions shown above: *APOE* ɛ4 (*n* = 413), education (*n* = 18), marital status (*n* = 13), depressive symptoms (*n* = 193), hypertension (*n* = 39), diabetes (*n* = 56), total cholesterol (*n* = 61), body mass index (*n* = 17), smoking (*n* = 7), and drinking (*n* = 8). Multiple imputation chained equations were applied to all missing covariates prior to modeling. SI conversion: To convert cholesterol to millimoles per liter, multiply by 0.0259.

Abbreviation: *APOE*, apolipoprotein E; ARIC, Atherosclerosis Risk in Communities

^a^ Not working outside the home including participants who identified as retired and no longer working for pay, homemaking or unemployed at Visit 2.

^b^ Not married including participants who identified as being divorced, separated, widowed, or never married at Visit 2.

^c^ Depressive symptoms scored using Maastricht Vital Exhaustion Questionnaire.

### 3.2 Mid‐life social relationships as potentially protective for dementia risk

**FIGURE 3:**
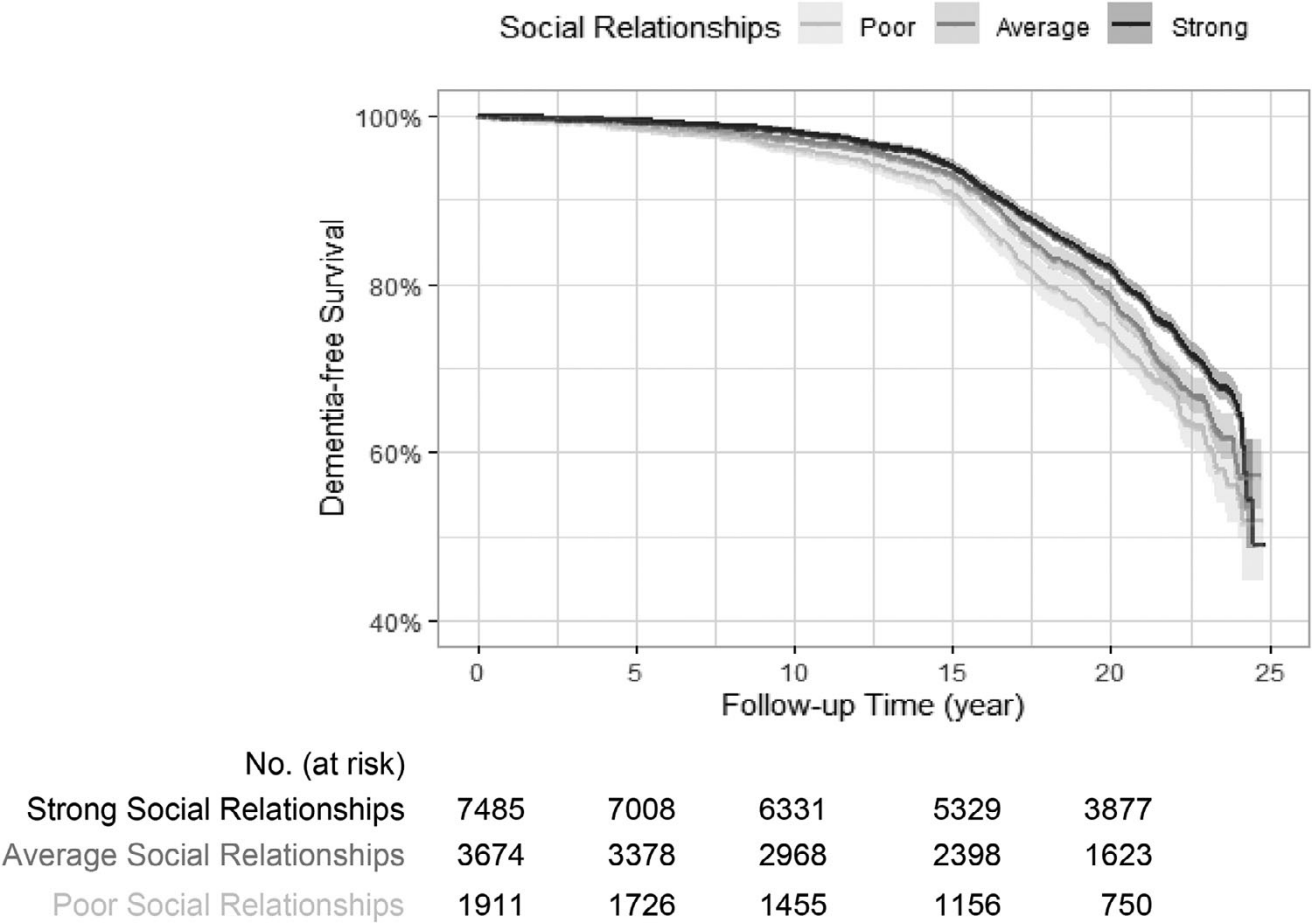
Kaplan–Meier curves of dementia‐free survival by mid‐life social relationships. Follow‐up time began 5 years following Atherosclerosis Risk in Communities (ARIC) Visit 2 when psychosocial factors were measured. Rates of dementia incidence differed significantly (log‐rank *p *< 0.001) in participants with varying levels of social relationships.

Across models, having strong (HR: 0.77, 95% CI: 0.69–0.86) or average social relationships (HR: 0.85, 95% CI: 0.75–0.96) in mid‐life was associated with a lower risk for developing dementia, compared to participants with poor social relationships in mid‐life (Table 2). Kaplan–Meier estimates of late‐life dementia risk stratified by mid‐life social relationships are shown (Figure 3).

**TABLE 2:** Adjusted hazard ratios for association of mid‐life social relationships with dementia risk (*N* = 13,070).

Social relationships	Model 1^a^ HR (95% CI)	Model 2^b^ HR (95% CI)	Model 3^c^ HR (95% CI)
Strong (*n* = 7458)	0.71(0.64–0.79)^**^	0.77(0.69–0.86)^**^	0.76(0.67–0.85)^**^
Average (*n* = 3674)	0.81 (0.72–0.91)^**^	0.85 (0.75–0.96)^**^	0.84 (0.74–0.95)^**^
Poor (*n* = 1911)	Reference	Reference	Reference
Age (per 1 year)	1.16 (1.15–1.17)^**^	1.15 (1.14–1.16)^**^	1.15 (1.14–1.16)^**^
Race‐ARIC Center			
White – Minnesota	1.07 (0.95–1.20)	1.11 (0.99–1.25)	1.09 (0.97–1.23)
White – Maryland	1.10 (0.99–1.24)	1.12 (1.00–1.25)	1.08 (0.97–1.22)
Black – Forsyth	1.29 (0.98–1.70)	1.24 (0.94–1.63)	1.10 (0.83–1.45)
Black – Jackson	1.65 (1.46–1.86)^**^	1.61 (1.43–1.81)^**^	1.41 (1.24–1.60)^**^
White – Forsyth	Reference	Reference	Reference
Sex (Men)	1.06 (0.98–1.14)	1.16 (1.06–1.26)^**^	1.14 (1.04–1.25)^**^
*APOE* ɛ4 carrier	1.88 (1.74–2.04)^**^	1.89 (1.75–2.05)^**^	1.90 (1.76–2.06)^**^
Education level			
Less than high school	Reference	Reference	Reference
High school or equivalent	0.76 (0.68–0.85)^**^	0.78 (0.70–0.87)^**^	0.80 (0.72–0.89)^**^
More than high school	0.64 (0.58–0.71)^**^	0.67 (0.61–0.74)^**^	0.70 (0.63–0.77)^**^
Not working outside the home	–	1.15 (1.06–1.26)^**^	1.13 (1.04–1.23)^**^
Not married	–	1.17 (1.06–1.29)^**^	1.15 (1.04–1.27)^**^
Depressive symptoms	–	1.21 (1.11– 1.32)^**^	1.18 (1.08–1.28)^**^
Hypertension	–	–	1.16 (1.07–1.26)^**^
Diabetes	–	–	1.53 (1.37–1.71)^**^
Body mass index (≥30 kg/m^2^)	–	–	1.12 (1.03–1.23)^*^
Total cholesterol (≥200 mg/dL)	–	–	1.03 (0.95–1.12)
Ever smoker	–	–	1.12 (1.03–1.22)^*^
Ever drinker	–	–	0.92 (0.83–1.02)

*Note*: SI conversion: To convert cholesterol to millimoles per liter, multiply by 0.0259.

Abbreviations: *APOE*, apolipoprotein E; HR, hazard ratio.

^a^ Model adjusted for age, race‐center, sex, *APOE* ɛ4, education.

^b^ Model adjusted for Model 1 covariates, occupational/marital status, and (having) depressive symptoms as measured at Visit 2.

^c^ Model adjusted for Model 2 covariates and vascular risk factors as measured at Visit 2.

* *P* ≤ 0.05

** *P* ≤ 0.01.

Our results did not show statistically significant evidence of effect modification by sex (*p‐*interaction = 0.14) or race (*p‐*interaction = 0.69). Stratified sex and race models are shown (Tables S1 and S2). Exploratory analyses that examined whether age (dichotomized at the sample median at Visit 2), *APOE* ɛ4 status, presence versus absence of depressive symptoms, or educational level modified the association between mid‐life social relationships and dementia were not statistically significant. Due to our prespecified analytic plan to evaluate effect modication by such factors, we further examined model findings from stratified groups to observe potential between‐group differences. Most notably, stronger associations between mid‐life social relationships and dementia risk were shown in participants **≥**57 years old (the sample median at Visit 2; Tables S3–S6).

### 3.3 Sensitivity analyses

Models assessing independent associations between mid‐life measures of social support and social isolation (as assessed via the ISEL‐SF and LSNS, respectively) with incident dementia risk are shown (Tables S7 and S8). In models assessing social support, participants with high and intermediate mid‐life social support had a lower late‐life risk of dementia, relative to those with low mid‐life social support. In models assessing social isolation across four levels, participants with low or moderate mid‐life risk of social isolation had a lower late‐life risk of dementia relative to those who were isolated in mid‐life, yet the association was not significant in those at high risk for isolation. Models excluding participants whose global cognitive function fell in the lowest fifth percentile at Visit 2 or those who had a stroke before the end of follow‐up showed results very similar to the primary models (Tables S9 and S10). Analyses, which accounted for death as a competing risk, showed results similar to the primary models, although the potentially protective effect conferred from mid‐life social relationships was attenuated (Table S11).

## 4 DISCUSSION

Results from this community‐based cohort study provide evidence that stronger social relationships in mid‐life are associated with a lower risk of incident dementia in late‐life. Findings remained robust to adjustments for demographics, *APOE* ɛ4, depressive symptoms, occupational status, marital status, and vascular risk factors measured in mid‐life. Secondary and exploratory analyses illustrated no significant effect modification by sex, race, or other examined factors (age, *APOE* ɛ4 status,,  depressive symptoms, or educational level).

Our findings build upon previous studies that have shown the potentially protective effect of social relationships on outcomes, such as sustained cognitive function and/or reduced dementia risk ^4^, ^5^, ^7^, ^10^, ^11^, ^13^, ^21^ This study significantly adds to existing literature in several ways. First, we examined social relationships in middle‐aged adults with carefully adjudicated dementia diagnoses over a long follow‐up period (median follow‐up: 19.3 years). Second, the design of our longitudinal study, which purposefully excluded any adults with dementia at baseline, and imposed an additional lag time of 5 years following the measurement of psychosocial health, helps account for reverse causation. Next, our examination of the association between mid‐life social relationships with late‐life dementia as the outcome of interest, in contrast to studies examining the rate of cognitive decline, is an important distinction. Incident dementia, as the outcome, highlights the development of a health condition rather than an interindividual rate of cognitive decline, which is highly dependent upon baseline cognition. Finally, we want to highlight that the cumulative incidence of dementia in this sample (20%) is aligned with global estimates of dementia for this age group.^41^ This is worth noting as it provides compelling evidence that our sample is not healthier or at lower risk for dementia than the general population, a common shortcoming in cohort studies.

Investigating social relationships in mid‐life relative to dementia in later life is particularly important to intervention and prevention efforts.^21^, ^30^ Mid‐life represents a significant window when lifestyle choices, including social behaviors, can be altered and prevention is still possible. In accordance with theories of cognitive reserve, the “buffering hypothesis” and related conceptual frameworks, engaging in social, physical, and intellectually stimulating activities in mid‐life may help instill habits of a healthy lifestyle that can be translated into later life, and furthermore, help sustain higher cognitive capabilities in elderly adults.^1^, ^42^, ^43^ Likewise, being socially active in mid‐life can confer simultaneous health benefits (e.g., adults with greater social contact are more likely to have rides to doctor appointments, prescription pick‐ups, company to go for a walk, etc).^44^, ^45^, ^46^

Effect modification by neither sex nor race was statistically significant in this sample. However, results indicate that mid‐life social relationships may be more beneficial in women and White participants relative to men and Black participants, respectively. Previous studies looking at varying aspects of psychosocial health have suggested that women may derive greater benefits of strong psychosocial health,^15^, ^25^ but this continues to be studied. Taken together, it is possible that these findings reflect that relationships and emotional connections are internalized differently in specified sex and racial groups, although this interpretation is speculative. Continuing to explore whether strong social relationships confer a greater protective effect in women and Black participants could be particularly significant, as both these groups are considered at an increased risk of dementia.^47^, ^48^ Likewise, related studies investigating sex and race differences are needed to support both intervention efforts and diversity, equity, and inclusion (DEI) research. These efforts are critical as researchers strive to better understand individualized risk profiles for dementia.^49^

Relatedly, the ARIC cohort encompasses a unique group of individuals in whom to study mid‐life social relationships in association with dementia risk. The median age of ARIC study participants when psychosocial health was assessed was 57 years old. This evaluation occurred in the early 1990s—a period that marked the introduction of home internet services, which may have influenced social relationships and perceptions of human connectedness. Many ARIC participants were born in the years around the time of the Great Depression and entered adulthood during pivotal events, such as World War II as well as the Korean and Vietnam wars. Racial desegregation further characterized this era and may have largely impacted Black ARIC participants, who were predominantly recruited from two field sites in the South (Jackson, MS and Winston‐Salem, NC). Altogether, this culmination of historical events in addition to societal changes that accompanied the subsequent technological revolution may have shaped psychosocial factors and the worldview of the cohort examined. Thus, our findings of a potentially protective effect of mid‐life social relationships in individuals who collectively lived through these events in both urban and rural communities further speaks to the powerful influence this risk factor may have on incident dementia.

Exploratory analyses in this cohort did not show significant interaction effects when examining factors such as age, *APOE* ɛ4, depressive symptoms, and education. Although we subsequently conducted stratified analyses, we want to caution against overinterpreting such findings. Our decision to analyze factors related to multiple activity domains (e.g., mental, cognitive, and psychosocial health) despite no evidence of effect modification stems from the scarcity of literature examining how theco‐existing nature and interactions between such characteristics may shape long‐term health outcomes.^22^

Sensitivity analyses illustrated that the potentially protective effect observed when measuring social relationships in mid‐life was consistent with that observed when examining social support and the risk of isolation. This provides additional evidence as to the validity of our social relationships measure, previously used in other studies of the ARIC cohort.^29^, ^50^ Notably, associations between social support or risk of social isolation with global cognitive decline in late‐life were not seen in a previous ARIC study, which measured cognition through use of an abbreviated cognitive battery.^19^ Use of a cognitive battery may not reflect ongoing disease development as well as evaluating incident dementia, which further incorporates functional status and informant interviews.

A complex behavior like psychosocial health may be difficult to quantify through means of questionnaires. The rationale that led us to examine social relationships as a composite measure was based upon the idea that social support and social isolation are independent constructs with underlying similarities.^31^, ^51^ The ISEL‐SF is thought to measure perceived social support, which has been shown to vary little over time, and focuses on the support that participants feel they are receiving with little emphasis on reciprocated social support.^31^ The LSNS is largely objective and captures many aspects of one's social network including social support, relationship closeness with friends and family, and aspects of loneliness, all of which may vary situationally.^31^ Both scales have a collective emphasis on relationships closeness with friends and family, with a lesser focus on how individuals see themselves and their role within their greater community. These measures capture many facets of psychosocial health but are not absolute. Beyond our efforts to include adjacent lifestyle factors, such as *APOE* e4 status, depressive symptoms, and educational level, we are hopeful that future work can additionally evaluate robust measures of psychosocial health taken at multiple time points throughout the life course in addition to other social determinants of health.

There are limitations to the present study. Questionnaires in this study were administered solely at Visit 2, which precluded us from measuring changes in psychosocial health as individuals aged from their 50s through 70s. Notably, previous studies have shown social relationships, and scores on these self‐reported scales do not largely fluctuate during this period of life.^31^, ^52^ We further acknowledge the possibility that some individuals may have experienced an earlier preclinical cognitive decline that influenced the psychosocial factors measured at Visit 2. To avoid this form of reverse causation, we added the 5‐year lag period following Visit 2 to eliminate any individuals who were already very close to having clinical symptoms of dementia at the time psychosocial factors were assessed. We further included a sensitivity analysis, which excluded the bottom 5% of individuals displaying low global cognition at Visit 2 in hopes to avoid the inclusion of individuals who were already in early stages of dementia. Despite these adjustments, survival bias still may have influenced our findings, as those with the poorest social relationships and cognitive function are less likely to be able to come in for study visits. However, having dementia events further identified by phone screening, hospitalization, and death ascertained via International Classification of Diseases (ICD) codes helps address this concern. Finally, we want to reiterate that although these findings were shown in a substantial sample of Black participants, they are not generalizable beyond Black and White participants recruited from within ARIC communities.

In conclusion, the present study illustrates that social relationships in mid‐life may have a potentially protective effect on dementia risk in older adults. In addition to research efforts that aim to reduce dementia risk proactively, emphasis on protective risk factors in mid‐life may serve as an encouraging, non‐pharmacological alternative for prevention efforts. Further evaluation of such factors in parallel to disease processes and disease‐modifying treatments is necessary to study aging and multifactorial diseases, such as dementia.

## CONFLICT OF INTEREST STATEMENT

The authors declare no conflicts of interest. Author disclosures are available in the Supporting Information.

## ETHICS STATEMENT AND CONSENT

The study was performed in accordance with the ethical standards as laid down in the 1964 Declaration of Helsinki and its later amendments or comparable ethical standards. All the institutional review boards of the respective institutions approved the Atherosclerosis Risk in Communities (ARIC) study, and participants signed informed consent for their involvement. Diversity, equity, and inclusion (DEI) was addressed in the study design, execution, and interpretation. Participants came from multiple study centers across the United States characterized by varying economic and racial profiles. When analyzing and interpreting the results, the study accounted for differences in age, sex, and race.

## Supporting information




Supporting File 1

Supporting File 2
