# Supplementary material for: Associations between mid‐life social relationships and the risk of incident dementia: The ARIC study
Source: Alzheimers Dement. 2025 Jul 14;21(7):e70365. doi: 10.1002/alz.70365 (PMC12260128; doi:10.1002/alz.70365)
Supplement: Supplementary file 1 — Supporting File 1 [file ALZ-21-e70365-s002.docx]

**Supplemental Figures and Tables**

**eFigure 1. ARIC Study Timeline**

**eFigure 2. Interpersonal Support Evaluation List-Short Form (ISEL-SF)**

**eFigure 3. The Lubben Social Network Scale (LSNS)**

**eTable 1. Adjusted Hazard Ratios for Association of Mid-Life Social Relationships with Dementia Risk, Stratified by Sex (n = 13070)**

**eTable 2. Adjusted Hazard Ratios for Association of Mid-Life Social Relationships with Dementia Risk, Stratified by Race (n = 13070)**

**eTable 3. Adjusted Hazard Ratios for Association of Mid-Life Social Relationships with Dementia Risk, Stratified by Mean Age at Visit 2 (n = 13070)**

**eTable 4. Adjusted Hazard Ratios for Association of Mid-Life Social Relationships with Dementia Risk, Stratified by *APOE***ε**4 status (n = 13070)**

**eTable 5. Adjusted Hazard Ratios for Association of Mid-Life Social Relationships with Dementia Risk, Stratified by Depressive Symptoms (n = 13070)**

**eTable 6. Adjusted Hazard Ratios for Association of Mid-Life Social Relationships with Dementia Risk, Stratified by Education Level (n = 13070)**

**eTable 7. Adjusted Hazard Ratios for Association of Mid-Life Social Support with Dementia Risk (n = 13070)**

**eTable 8. Adjusted Hazard Ratios for Association of Mid-Life Social Isolation with Dementia Risk (n = 13070)**

**eTable 9. Adjusted Hazard Ratios for Association of Mid-Life Social Relationships with Dementia Risk, Excluding those with Global Cognition in Lowest 5^th^ Percentile at Visit 2 (n = 12448)**

**eTable 10. Adjusted Hazard Ratios for Association of Mid-Life Social Relationships with Dementia Risk, Excluding those who had Incident Stroke before End of Follow-Up (n = 11708)**

**eTable 11. Adjusted Sub-Hazard Ratios for Association of Mid-Life Relationships with Dementia Risk, Accounting for Competing Risk of Death (n = 13070)**

**eFigure 1. ARIC Study Timeline**

**
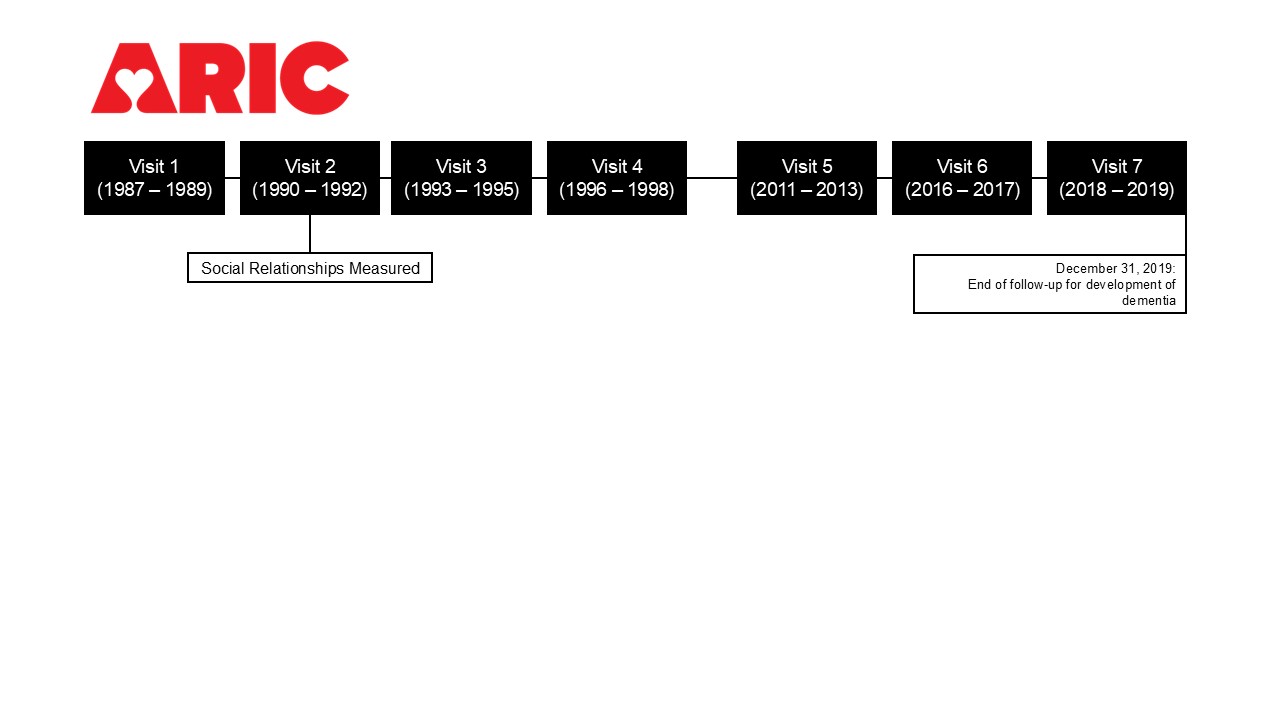
**

NOTE: Study timeline *only* includes study visits from which data were used in the present analysis.

**eFigure 2. Interpersonal Support Evaluation List-Short Form (ISEL-SF)**

| **No.** | **Questions** | **Responses** | | | |
| --- | --- | --- | --- | --- | --- |
|  |  | **Definitely true** | **Probably true** | **Probably false** | **Definitely**  **false** |
| **Appraisal support** | | **Scores** | | | |
| 1. | There really is no one who can give me an objective view of how I’m handling my problems. | 0 | 1 | 2 | 3 |
| 2. | When I need suggestions on how to deal with a personal problem, I know someone I can turn to. | 3 | 2 | 1 | 0 |
| 3. | There is really no one I can trust to give me good financial advice. | 0 | 1 | 2 | 3 |
| 4. | There is at least one person I know whose advice I really trust. | 3 | 2 | 1 | 0 |
| **Self-esteem** | |  |  |  |  |
| 5. | Most of my friends are more interesting than I am. | 0 | 1 | 2 | 3 |
| 6. | Most of my friends are more successful at making changes in their lives than I am. | 0 | 1 | 2 | 3 |
| 7. | I am more satisfied with my life than most people are with theirs. | 3 | 2 | 1 | 0 |
| 8. | I have a hard time keeping pace with my friends. | 0 | 1 | 2 | 3 |
| **Belonging support** | |  |  |  |  |
| 9. | When I feel lonely, there are several people I can talk to. | 3 | 2 | 1 | 0 |
| 10. | I often meet or talk with family or friends. | 3 | 2 | 1 | 0 |
| 11. | I feel like I am not always included by my circle of friends. | 0 | 1 | 2 | 3 |
| 12. | I don’t often get invited to do things with others. | 0 | 1 | 2 | 3 |
| **Tangible support** | |  |  |  |  |
| 13. | If I were sick and needed someone (friend, family member, or acquaintance) to take me to the doctor, I would have trouble finding someone. | 0 | 1 | 2 | 3 |
| 14. | If I were sick, I could easily find someone to help me with my daily chores. | 3 | 2 | 1 | 0 |
| 15. | If I had to go out of town for a few weeks, it would be difficult to find someone who would look after my house or apartment (the plants, pets, garden, etc.). | 0 | 1 | 2 | 3 |
| 16. | It would be difficult to find someone who would lend me their car for a few hours. | 0 | 1 | 2 | 3 |

NOTE: Form filled out by participant during ARIC Visit 2 with pen and paper. Order shown above lists statement by each subscale and is not reflective of the order of the questionnaire given at visit 2. Participants instructed not to leave any questions blank unless instructed to skip to another question.

**eFigure 3. The Lubben Social Network Scale (LSNS)**

| **No.** | **Questions** | **Scores** | | | | | |
| --- | --- | --- | --- | --- | --- | --- | --- |
|  |  | 0 | 1 | 2 | 3 | 4 | 5 |
|  |  | **Responses** | | | | | |
| 1. | How many relatives do you see or hear from at least once a month? | 0 | 1 | 2 | 3-4 | 5-8 | ≥9 |
| 2. | How many relatives do you feel close to? That is, how many of them do you feel at ease with, can talk to about private matters, or can call on for help? |  |  |  |  |  |  |
| 3. | Do you have any close friends? That is, do you have any friends with whom you feel at ease with, can talk to about private matters, or can call on for help? If so, how many? |  |  |  |  |  |  |
| 4. | How many of these friends do you see or hear from at least once a month? |  |  |  |  |  |  |
| 5. | Tell me about the relative with whom you have the most contact: How often do you see or hear from that person? | Less than monthly | Monthly | A few times  a month | Weekly | A few times  a week | Daily |
| 6. | Tell me about the friend with whom you have the most contact. How often do you see or hear from that person? |  |  |  |  |  |  |
| 7. | When you have an important decision to make, how frequently do you have someone you can talk to about it? | Never | Seldom | Some-  times | Often | Very often | Always |
| 8. | When other people you know have an important decision to make, how frequently do they talk to you about it? |  |  |  |  |  |  |
| 9a. | Does anybody rely on you to something for them each day? E.g.: shopping, making dinner, doing repairs, cleaning house providing childcare, etc.? | - | - | - | - | - | Yes |
| 9b. | How frequently do you help anybody with things like shopping, filling out forms, doing repairs, providing childcare, etc.? | Never | Seldom | Some-  times | Often | Very often | - |
| 10. | Do you live alone or with other people? | Live alone | Live with other unrelated individuals  (one point) | | Live with other relative or friends (four points) | | Live with spouse |

NOTE: Form filled out by participant during ARIC visit 2 with pen and paper. Order shown above is grouped by scoring and not reflective of the order of the questionnaire given at visit 2. Participants are instructed not to leave any questions blank unless instructed to skip to another question.

**eTable 1. Adjusted Hazard Ratios for Association of Mid-Life Social Relationships with Dementia Risk, Stratified by Sex (n = 13070)**

|  | **Men (n = 5802)** | | | **Women (n = 7268)** | | |
| --- | --- | --- | --- | --- | --- | --- |
| **Social Relationships** | **Model 1^a^ HR**  **(95% CI)** | **Model 2^b^ HR**  **(95% CI)** | **Model 3^c^ HR**  **(95% CI)** | **Model 1^a^ HR**  **(95% CI)** | **Model 2^b^ HR**  **(95% CI)** | **Model 3^c^ HR**  **(95% CI)** |
| Strong | 0.75  (0.64 – 0.89)** | 0.82  (0.69 – 0.97)* | 0.80  (0.68 – 0.95)* | 0.67  (0.58 - 0.78)** | 0.73  (0.62 – 0.85)** | 0.72  (0.61 – 0.84)** |
| Average | 0.92  (0.77 – 1.09) | 0.96  (0.81 – 1.15) | 0.95  (0.79 – 1.14) | 0.73  (0.62 – 0.86)** | 0.76  (0.65 – 0.90)** | 0.76  (0.64 – 0.89)** |
| Poor | Reference | Reference | Reference | Reference | Reference | Reference |
| **Age**  **(per 1 year)** | 1.15  (1.14 - 1.17)** | 1.15  (1.13 – 1.16)** | 1.15  (1.13 - 1.16)** | 1.17  (1.16 – 1.18)** | 1.16  (1.15 – 1.17)** | 1.16  (1.14 – 1.17)** |
| **Race-ARIC Center** |  |  |  |  |  |  |
| White - Minnesota | 1.05  (0.89 – 1.25) | 1.07  (0.90 – 1.27) | 1.06  (0.89 – 1.26) | 1.09  (0.93 – 1.28) | 1.14  (0.97 – 1.34) | 1.12  (0.95 – 1.33) |
| White – Maryland | 0.95  (0.80 – 1.14) | 0.95  (0.80 – 1.14) | 0.94  (0.78 – 1.12) | 1.25  (1.08 – 1.45)** | 1.26  (1.09 – 1.46)** | 1.22  (1.05 – 1.42)* |
| Black – Forsyth | 1.35  (0.84 – 2.16) | 1.34  (0.84 – 2.14) | 1.26  (0.79 – 2.03) | 1.28  (0.91 – 1.79) | 1.22  (0.87 – 1.72) | 1.06  (0.75 – 1.50) |
| Black - Jackson | 1.72  (1.42 – 2.08)** | 1.68  (1.39 – 2.04)** | 1.54  (1.27 – 1.88)** | 1.63  (1.40 – 1.90)** | 1.59  (1.37 – 1.86)** | 1.38  (1.17 – 1.62)** |
| White - Forsyth | Reference | Reference | Reference | Reference | Reference | Reference |
| ***APOE***ε**4 Carrier** | 1.71  (1.51 – 1.94)** | 1.72  (1.52 – 1.94)** | 1.73  (1.53 – 1.96)** | 2.04  (1.83 – 2.26)** | 2.05  (1.84 – 2.27)** | 2.06  (1.85 – 2.28)** |
| **Education Level** |  |  |  |  |  |  |
| Less than high school | Reference | Reference | Reference | Reference | Reference | Reference |
| High school or equivalent | 0.87  (0.73 – 1.04) | 0.90  (0.75 – 1.07) | 0.91  (0.76 – 1.08) | 0.69  (0.60 – 0.79)** | 0.71  (0.62 – 0.81)** | 0.73  (0.63 – 0.83)** |
| More than high school | 0.69  (0.59 – 0.81)** | 0.72  (0.62 – 0.85)** | 0.75  (0.64 – 0.88)** | 0.60  (0.52 – 0.68)** | 0.63  (0.55 – 0.72)** | 0.66  (0.57 – 0.75)** |
| **Not working outside the home** | ----- | 1.08  (0.94 – 1.25) | 1.06  (0.92 – 1.22) | ----- | 1.18  (1.06 – 1.32)** | 1.16  (1.04 – 1.30)* |
| **Not Married** | ----- | 1.16  (0.95 – 1.41) | 1.16  (0.95 – 1.41) | ----- | 1.18  (1.05 – 1.33)** | 1.16  (1.03 - 1.30)* |
| **Depressive Symptoms** | ----- | 1.30  (1.12– 1.51)** | 1.25  (1.08 – 1.46)** | ----- | 1.16  (1.04 – 1.30)** | 1.14  (1.02 – 1.28)* |
| **Hypertension** | ----- | ----- | 1.15  (1.01 – 1.31)* | ----- | ----- | 1.18  (1.06 – 1.32)** |
| **Diabetes** | ----- | ----- | 1.71  (1.46 – 2.02)** | ----- | ----- | 1.40  (1.21 – 1.63)** |
| **Body Mass Index**  **(≥ 30 kg/m^2^)** | ----- | ----- | 1.15  (1.00 – 1.33)* | ----- | ----- | 1.10  (0.98 – 1.24) |
| **Total Cholesterol**  **(≥ 200 mg/dL)** | ----- | ----- | 0.97  (0.86 – 1.10) | ----- | ----- | 1.08  (0.97 – 1.21) |
| **Ever smoker** | ----- | ----- | 1.15  (1.00 – 1.32)* | ----- | ----- | 1.11  (1.00 – 1.24) |
| **Ever drinker** | ----- | ----- | 0.85  (0.71 – 1.03) | ----- | ----- | 0.94  (0.84 – 1.06) |

**P* ≤ 0.05; ***P* ≤ 0.01; Abbreviations: *APOE* = apolipoprotein E; HR = hazard ratio. SI conversion: To convert cholesterol to millimoles per liter, multiply by 0.0259. Effect modification of sex on the association between mid-life social relationships and dementia risk was not significant (model 2 *p*-interaction = 0.14).

^a^Model adjusted for age, race-center, *APOE*ε4, education

^b^Model adjusted for model 1 covariates, occupational/marital status and (having) depressive symptoms at visit 2

^c^Model adjusted for model 2 covariates and vascular risk factors as measured at visit 2

**eTable 2. Adjusted Hazard Ratios for Association of Mid-Life Social Relationships**

**with Dementia Risk, Stratified by Race (n = 13070)**

|  | **Black (n = 3127)** | | | **White (n = 9943)** | | |
| --- | --- | --- | --- | --- | --- | --- |
| **Social Relationships** | **Model 1^a^ HR**  **(95% CI)** | **Model 2^b^ HR**  **(95% CI)** | **Model 3^c^ HR**  **(95% CI)** | **Model 1^a^ HR**  **(95% CI)** | **Model 2^b^ HR**  **(95% CI)** | **Model 3^c^ HR**  **(95% CI)** |
| Strong | 0.76  (0.62 – 0.93)** | 0.85  (0.69 – 1.05) | 0.82  (0.66 – 1.01) | 0.69  (0.60 – 0.78)** | 0.74  (0.64 – 0.84)** | 0.73  (0.64 – 0.84)** |
| Average | 0.90  (0.73 – 1.12) | 0.95  (0.76 – 1.18) | 0.94  (0.75 – 1.17) | 0.78  (0.68 – 0.90)** | 0.81  (0.70 – 0.93)** | 0.80  (0.69 – 0.93)** |
| Poor | Reference | Reference | Reference | Reference | Reference | Reference |
| **Age**  **(per 1 year)** | 1.13  (1.11 – 1.14)** | 1.12  (1.11 – 1.14)** | 1.12  (1.10 – 1.14)** | 1.18  (1.16 – 1.19)** | 1.17  (1.16 – 1.18)** | 1.17  (1.16 – 1.18)** |
| **Sex (Men)** | 1.16  (1.00 – 1.35) | 1.31  (1.11 – 1.54)** | 1.39  (1.17 – 1.66)** | 1.02  (0.93 – 1.13) | 1.11  (1.01 – 1.23)* | 1.06  (0.96 – 1.18) |
| ***APOE***ε**4 Carrier** | 1.50  (1.30 – 1.74)** | 1.50  (1.30 – 1.74)** | 1.51  (1.30 – 1.75)** | 2.08  (1.89 – 2.28)** | 2.09  (1.90 – 2.29)** | 2.11  (1.92 – 2.31)** |
| **Education Level** |  |  |  |  |  |  |
| Less than high school | Reference | Reference | Reference | Reference | Reference | Reference |
| High school or equivalent | 0.70  (0.58 – 0.86)** | 0.74  (0.61 – 0.90)** | 0.74  (0.60 – 0.90)** | 0.78  (0.69 – 0.89)** | 0.80  (0.71 – 0.91)** | 0.83  (0.73 – 0.95)** |
| More than high school | 0.53  (0.45 – 0.62)** | 0.56  (0.47 – 0.67)** | 0.57  (0.48 – 0.68)** | 0.68  (0.60 – 0.77)** | 0.71  (0.63 – 0.81)** | 0.75  (0.66 – 0.85)** |
| **Not working outside the home** | ----- | 1.13  (0.96 – 1.33) | 1.09  (0.93 – 1.29) | ----- | 1.14  (1.03 – 1.27)** | 1.12  (1.01 – 1.24)* |
| **Not Married** | ----- | 1.12  (0.96 – 1.31) | 1.12  (0.96 – 1.31) | ----- | 1.20  (1.06 – 1.37)** | 1.19  (1.04 – 1.35)** |
| **Depressive Symptoms** | ----- | 1.36  (1.16 – 1.60)** | 1.33  (1.14 – 1.57)** | ----- | 1.15  (1.03 – 1.28)* | 1.11  (1.00 – 1.23) |
| **Hypertension** | ----- | ----- | 1.16  (1.00 – 1.34) | ----- | ----- | 1.16  (1.05 – 1.28)** |
| **Diabetes** | ----- | ----- | 1.47  (1.24 – 1.74)** | ----- | ----- | 1.62  (1.41 – 1.87)** |
| **Body Mass Index**  **(≥ 30 kg/m^2^)** | ----- | ----- | 0.94  (0.81 – 1.10) | ----- | ----- | 1.24  (1.11 – 1.38)** |
| **Total Cholesterol**  **(≥ 200 mg/dL)** | ----- | ----- | 0.93  (0.80 – 1.07) | ----- | ----- | 1.09  (0.99 – 1.20) |
| **Ever smoker** | ----- | ----- | 0.93  (0.79 – 1.09) | ----- | ----- | 1.19  (1.07 – 1.31)** |
| **Ever drinker** | ----- | ----- | 0.92  (0.78 – 1.08) | ----- | ----- | 0.91  (0.81 – 1.03) |

**P* ≤ 0.05; ***P* ≤ 0.01; Abbreviations: *APOE* = apolipoprotein E; HR = hazard ratio. SI conversion: To convert cholesterol to millimoles per liter, multiply by 0.0259. Effect modification of race on the association between mid-life social relationships and dementia risk was not significant (model 2 *p*-interaction = 0.69).

^a^Model adjusted for age, sex, *APOE*ε4, education

^b^Model adjusted for model 1 covariates, occupational/marital status and (having) depressive symptoms at visit 2

^c^Model adjusted for model 2 covariates and vascular risk factors measured at visit 2

**eTable 3. Adjusted Hazard Ratios for Association of Mid-Life Social Relationships with Dementia Risk; Stratified by Median Age at Visit 2 (n = 13070)**

|  | **Age <57 years old (n = 6494)** | | | **Age ≥ 57 years old (n = 6576)** | | |
| --- | --- | --- | --- | --- | --- | --- |
| **Social Relationships** | **Model 1^a^ HR**  **(95% CI)** | **Model 2^b^ HR**  **(95% CI)** | **Model 3^c^ HR**  **(95% CI)** | **Model 1^a^ HR**  **(95% CI)** | **Model 2^b^ HR**  **(95% CI)** | **Model 3^c^ HR**  **(95% CI)** |
| Strong | 0.76  (0.63 – 0.93)** | 0.87  (0.71 – 1.07) | 0.87  (0.71 – 1.07) | 0.74  (0.65 – 0.84)** | 0.80  (0.70 – 0.92)** | 0.79  (0.68 – 0.90)** |
| Average | 0.94  (0.76 – 1.16) | 1.00  (0.81 – 1.24) | 1.00  (0.81 – 1.23) | 0.80  (0.70 – 0.93)** | 0.85  (0.74 – 0.99)* | 0.84  (0.73 – 0.98)* |
| Poor | Reference | Reference | Reference | Reference | Reference | Reference |
| **Race-ARIC Center** |  |  |  |  |  |  |
| White - Minnesota | 1.15  (0.91 – 1.45) | 1.22  (0.97 – 1.55) | 1.21  (0.96 – 1.54) | 1.06  (0.92 – 1.21) | 1.11  (0.97 – 1.27) | 1.12  (0.97 – 1.29) |
| White – Maryland | 1.33  (1.06 – 1.67)* | 1.38  (1.10 – 1.73)** | 1.33  (1.06 – 1.68)* | 1.03  (0.91 – 1.18) | 1.06  (0.93 – 1.21) | 1.05  (0.92 – 1.20) |
| Black – Forsyth | 1.19  (0.70 - 2.04) | 1.20  (0.70 – 2.06) | 1.04  (0.60 – 1.79) | 1.33  (0.97 – 1.83) | 1.26  (0.92– 1.74) | 1.14  (0.82 – 1.57) |
| Black - Jackson | 2.10  (1.69 – 2.61)** | 2.14  (1.72 – 2.67)** | 1.74  (1.38 – 2.19)** | 1.30  (1.13 – 1.51)** | 1.27  (1.09 – 1.47)** | 1.15  (0.99 – 1.35) |
| White - Forsyth | Reference | Reference | Reference | Reference | Reference | Reference |
| **Sex (Men)** | 1.22  (1.06 – 1.41)** | 1.41  (1.22 – 1.64)** | 1.40  (1.20 – 1.63)** | 0.98  (0.89 – 1.08) | 1.12  (1.01 – 1.24)* | 1.12  (1.00 – 1.25)* |
| ***APOE***ε**4 Carrier** | 1.83  (1.58 – 2.11)** | 1.84  (1.59 – 2.12)** | 1.86  (1.61 – 2.15)** | 1.88  (1.71 – 2.07)** | 1.90  (1.73 – 2.10)** | 1.92  (1.74 – 2.12)** |
| **Education Level** |  |  |  |  |  |  |
| Less than high school | Reference | Reference | Reference | Reference | Reference | Reference |
| High school or equivalent | 0.60  (0.50 – 0.73)** | 0.65  (0.54 – 0.79)** | 0.67  (0.55 – 0.81)** | 0.70  (0.62 – 0.80)** | 0.74  (0.65 – 0.84)** | 0.75  (0.66 – 0.85)** |
| More than high school | 0.43  (0.35 – 0.51)** | 0.48  (0.40 – 0.58)** | 0.51  (0.42 – 0.61)** | 0.65  (0.57 – 0.73)** | 0.70  (0.62 – 0.79)** | 0.72  (0.64 – 0.81)** |
| **Not working outside the home** | ----- | 1.48  (1.25 – 1.76)** | 1.42  (1.20 – 1.69)** | ----- | 1.43  (1.30 – 1.57)** | 1.41  (1.28 – 1.55)** |
| **Not Married** | ----- | 1.06  (0.89 – 1.26) | 1.06  (0.89 – 1.27) | ----- | 1.33  (1.18 – 1.50)** | 1.31  (1.16 – 1.48)** |
| **Depressive Symptoms** | ----- | 1.43  (1.22– 1.67)** | 1.34  (1.15 – 1.57)** | ----- | 1.14  (1.02 – 1.27)* | 1.11  (1.00 – 1.24) |
| **Hypertension** | ----- | ----- | 1.39  (1.20 – 1.62)** | ----- | ----- | 1.14  (1.03 – 1.26)* |
| **Diabetes** | ----- | ----- | 1.85  (1.53 – 2.24)** | ----- | ----- | 1.44  (1.26 – 1.64)** |
| **Body Mass Index**  **(≥ 30 kg/m^2^)** | ----- | ----- | 1.11  (0.95 – 1.29) | ----- | ----- | 1.01  (0.91 – 1.13) |
| **Total Cholesterol**  **(≥ 200 mg/dL)** | ----- | ----- | 1.21  (1.05 – 1.39)** | ----- | ----- | 1.01  (0.92 – 1.11) |
| **Ever smoker** | ----- | ----- | 1.24  (1.06 – 1.44)** | ----- | ----- | 1.03  (0.93 – 1.15) |
| **Ever drinker** | ----- | ----- | 0.84  (0.70 – 1.00)* | ----- | ----- | 0.91  (0.81 – 1.03) |

**P* ≤ 0.05; ***P* ≤ 0.01; Abbreviations: *APOE* = apolipoprotein E; HR = hazard ratio. SI conversion: To convert cholesterol to millimoles per liter, multiply by 0.0259. Effect modification of age on the association between mid-life social relationships and dementia risk was not significant (model 2 *p*-interaction = 0.31).

^a^Model adjusted for sex, race-center, *APOE*ε4, education

^b^Model adjusted for model 1 covariates, occupational/marital status and (having) depressive symptoms at visit 2

^c^Model adjusted for model 2 covariates and vascular risk factors measured at visit 2

**eTable 4. Adjusted Hazard Ratios for Association of Mid-Life Social Relationships with Dementia Risk, Stratified by *APOE***ε**4 status (n = 13070)**

|  | **Has 1+ *APOE***ε**4 alleles (n = 4007)** | | | **No *APOE***ε**4 alleles (n = 9063)** | | |
| --- | --- | --- | --- | --- | --- | --- |
| **Social Relationships** | **Model 1^a^ HR**  **(95% CI)** | **Model 2^b^ HR**  **(95% CI)** | **Model 3^c^ HR**  **(95% CI)** | **Model 1^a^ HR**  **(95% CI)** | **Model 2^b^ HR**  **(95% CI)** | **Model 3^c^ HR**  **(95% CI)** |
| Strong | 0.73  (0.61 – 0.86)** | 0.76  (0.64 – 0.91)** | 0.73  (0.61 – 0.88)** | 0.70  (0.60 – 0.80)** | 0.78  (0.67 – 0.91)** | 0.78  (0.67 – 0.91)** |
| Average | 0.78  (0.65 – 0.94)** | 0.80  (0.66 – 0.96)* | 0.79  (0.66 – 0.96)** | 0.84  (0.71 – 0.98)* | 0.88  (0.75 – 1.04) | 0.88  (0.75 – 1.04) |
| Poor | Reference | Reference | Reference | Reference | Reference | Reference |
| **Age (per 1 year)** | 1.15  (1.14– 1.17)** | 1.15  (1.13 – 1.16)** | 1.15  (1.13 – 1.16)** | 1.17  (1.15 – 1.18)** | 1.16  (1.15 – 1.17)** | 1.16  (1.14 – 1.17)** |
| **Race-ARIC Center** |  |  |  |  |  |  |
| White - Minnesota | 1.18  (0.98 – 1.42) | 1.20  (1.00 – 1.45) | 1.23  (1.02 – 1.49)* | 1.00  (0.86 – 1.16) | 1.04  (0.89 – 1.21) | 0.99  (0.85 – 1.16) |
| White – Maryland | 1.15  (0.95 – 1.39) | 1.15  (0.95 – 1.39) | 1.17  (0.96 – 1.41) | 1.07  (0.92 – 1.23) | 1.09  (0.94 – 1.26) | 1.02  (0.88 – 1.19) |
| Black – Forsyth | 1.25  (0.83 – 1.88) | 1.23  (0.81 – 1.86) | 1.10  (0.72 – 1.67) | 1.34  (0.93 – 1.95) | 1.26  (0.87 – 1.82) | 1.10  (0.76 – 1.60) |
| Black - Jackson | 1.45  (1.20 – 1.74)** | 1.43  (1.18 – 1.73)** | 1.28  (1.05 – 1.56)* | 1.90  (1.62 – 2.22)** | 1.84  (1.57 – 2.15)** | 1.57  (1.33 – 1.85)** |
| White - Forsyth | Reference | Reference | Reference | Reference | Reference | Reference |
| **Sex (Men)** | 0.95  (0.84 – 1.07) | 0.99  (0.87 – 1.13) | 0.98  (0.85 – 1.13) | 1.16  (1.05 – 1.29)** | 1.32  (1.18 – 1.48)** | 1.31  (1.16 – 1.47)** |
| **Education Level** |  |  |  |  |  |  |
| Less than high school | Reference | Reference | Reference | Reference | Reference | Reference |
| High school or equivalent | 0.75  (0.63 – 0.89)** | 0.76  (0.64 – 0.90)** | 0.78  (0.65 – 0.92)** | 0.75  (0.66 – 0.86)** | 0.78  (0.68 – 0.89)** | 0.79  (0.69 – 0.91)** |
| More than high school | 0.72  (0.61 – 0.84)** | 0.74  (0.63 – 0.86)** | 0.77  (0.65 – 0.90)** | 0.58  (0.51 – 0.66)** | 0.61  (0.53 – 0.70)** | 0.63  (0.55 – 0.72)** |
| **Not working outside the home** | ----- | 1.11  (0.97 – 1.26) | 1.08  (0.95 – 1.24) | ----- | 1.19  (1.07 – 1.34)** | 1.17  (1.04 – 1.31)** |
| **Not Married** | ----- | 1.06  (0.91 – 1.24) | 1.03  (0.88 – 1.21) | ----- | 1.28  (1.12 – 1.46)** | 1.25  (1.10 – 1.43)** |
| **Depressive Symptoms** | ----- | 1.12  (0.97– 1.29) | 1.08  (0.94 – 1.25) | ----- | 1.28  (1.14 – 1.43)** | 1.24  (1.11 – 1.40)** |
| **Hypertension** | ----- | ----- | 1.16  (1.02 – 1.32)* | ----- | ----- | 1.16  (1.04 – 1.30)** |
| **Diabetes** | ----- | ----- | 1.51  (1.27 – 1.80)** | ----- | ----- | 1.55  (1.35 – 1.78)** |
| **Body Mass Index**  **(≥ 30 kg/m^2^)** | ----- | ----- | 1.02  (0.89 – 1.17) | ----- | ----- | 1.20  (1.07 – 1.35)** |
| **Total Cholesterol**  **(≥ 200 mg/dL)** | ----- | ----- | 1.04  (0.92 – 1.18) | ----- | ----- | 1.04  (0.94 – 1.15) |
| **Ever smoker** | ----- | ----- | 1.20  (1.05 – 1.37)** | ----- | ----- | 1.07  (0.95 – 1.19) |
| **Ever drinker** | ----- | ----- | 0.80  (0.69 –0.93)** | ----- | ----- | 1.02  (0.89 – 1.16) |

**P* ≤ 0.05; ***P* ≤ 0.01; Abbreviations: *APOE* = apolipoprotein E; HR = hazard ratio.

SI conversion: To convert cholesterol to millimoles per liter, multiply by 0.0259. Effect modification of *APOE*ε4 alleles on the association between mid-life social relationships and dementia risk was not significant (model 2 *p*-interaction = 0.35). Total N reflects data imputed for 413 participants.

^a^Model adjusted for age, race-center, sex, education

^b^Model adjusted for model 1 covariates, occupational/marital status and (having) depressive symptoms at visit 2

^c^Model adjusted for model 2 covariates and vascular risk factors measured at visit 2

**eTable 5. Adjusted Hazard Ratios for Association of Mid-Life Social Relationships with Dementia Risk, Stratified by Depressive Symptoms (n = 13070)**

|  | **Has Depressive Symptoms (n = 3932)** | | | **Does not have Depressive Symptoms (n = 9138)** | | |
| --- | --- | --- | --- | --- | --- | --- |
| **Social Relationships** | **Model 1^a^ HR**  **(95% CI)** | **Model 2^b^ HR**  **(95% CI)** | **Model 3^c^ HR**  **(95% CI)** | **Model 1^a^ HR**  **(95% CI)** | **Model 2^b^ HR**  **(95% CI)** | **Model 3^c^ HR**  **(95% CI)** |
| Strong | 0.73  (0.62 – 0.87)** | 0.76  (0.63 – 0.90)** | 0.73  (0.61 – 0.87)** | 0.77  (0.66 – 0.90)** | 0.79  (0.68 – 0.92)** | 0.77  (0.66 – 0.90)** |
| Average | 0.82  (0.69 – 0.97)* | 0.84  (0.70 – 1.00)* | 0.83  (0.70 – 0.99)* | 0.85  (0.71 – 1.00) | 0.87  (0.73 – 1.03) | 0.85  (0.72 – 1.01) |
| Poor | Reference | Reference | Reference | Reference | Reference | Reference |
| **Age (per 1 year)** | 1.13  (1.12– 1.15)** | 1.13  (1.11 – 1.14)** | 1.13  (1.11 – 1.14)** | 1.17  (1.16 – 1.18)** | 1.17  (1.16 – 1.18)** | 1.17  (1.16 – 1.18)** |
| **Race-ARIC Center** |  |  |  |  |  |  |
| White - Minnesota | 1.04  (0.83 – 1.30) | 1.06  (0.85– 1.33) | 1.09  (0.87 – 1.38) | 1.12  (0.98 – 1.29) | 1.14  (0.99 – 1.31) | 1.11  (0.96 – 1.28) |
| White – Maryland | 0.93  (0.76 – 1.13) | 0.94  (0.78 – 1.15) | 0.95  (0.78 – 1.16) | 1.23  (1.07 – 1.42)** | 1.24  (1.08 – 1.43)** | 1.20  (1.04 – 1.38)* |
| Black – Forsyth | 1.04  (0.68 – 1.60) | 1.02  (0.66 – 1.58) | 0.93  (0.60 – 1.44) | 1.54  (1.08 - 2.19)* | 1.50  (1.05 – 2.14)* | 1.33  (0.93 – 1.91) |
| Black - Jackson | 1.66  (1.37 – 2.02)** | 1.61  (1.32 – 1.96)** | 1.42  (1.16 – 1.75)** | 1.63  (1.40 – 1.89)** | 1.60  (1.37 – 1.87)** | 1.41  (1.20 – 1.65)** |
| White - Forsyth | Reference | Reference | Reference | Reference | Reference | Reference |
| **Sex (Men)** | 1.18  (1.01 – 1.37)* | 1.26  (1.08 – 1.48)** | 1.24  (1.05 – 1.47)* | 1.07  (0.97 – 1.18) | 1.11  (1.01 – 1.23)* | 1.10  (0.99 – 1.23) |
| ***APOE***ε**4 Carrier** | 1.65  (1.44 – 1.90)** | 1.66  (1.44 – 1.91)** | 1.67  (1.45 – 1.93)** | 2.02  (1.84 – 2.23)** | 2.02  (1.84 – 2.23)** | 2.03  (1.84 – 2.24)** |
| **Education Level** |  |  |  |  |  |  |
| Less than high school | Reference | Reference | Reference | Reference | Reference | Reference |
| High school or equivalent | 0.76  (0.64 – 0.90)** | 0.77  (0.65 – 0.92)** | 0.79  (0.67 – 0.94)** | 0.80  (0.69– 0.92)** | 0.80  (0.70 – 0.92)** | 0.81  (0.70 – 0.93)** |
| More than high school | 0.59  (0.49 – 0.70)** | 0.60  (0.51 – 0.72)** | 0.63  (0.53 – 0.75)** | 0.71  (0.62 – 0.81)** | 0.72  (0.63 – 0.82)** | 0.74  (0.65 – 0.84)** |
| **Not working outside the home** | ----- | 1.23  (1.06 – 1.42)** | 1.19  (1.03 – 1.37)* | ----- | 1.10  (0.99 – 1.22) | 1.09  (0.98 – 1.21) |
| **Not Married** | ----- | 1.20  (1.02 – 1.40)* | 1.17  (0.99 – 1.37) | ----- | 1.15  (1.02 – 1.31)* | 1.13  (1.00 – 1.29) |
| **Hypertension** | ----- | ----- | 1.23  (1.06 – 1.42)** | ----- | ----- | 1.11  (1.00 – 1.23) |
| **Diabetes** | ----- | ----- | 1.68  (1.41 – 1.99)** | ----- | ----- | 1.41  (1.22 – 1.63)** |
| **Body Mass Index**  **(≥ 30 kg/m^2^)** | ----- | ----- | 0.97  (0.84 – 1.13) | ----- | ----- | 1.23  (1.10– 1.37)** |
| **Total Cholesterol**  **(≥ 200 mg/dL)** | ----- | ----- | 0.98  (0.85 – 1.12) | ----- | ----- | 1.06  (0.96 – 1.17) |
| **Ever smoker** | ----- | ----- | 1.11  (0.96 – 1.29) | ----- | ----- | 1.12  (1.00 – 1.24)* |
| **Ever drinker** | ----- | ----- | 0.85  (0.73 – 1.00)* | ----- | ----- | 0.95  (0.84 – 1.09) |

**P* ≤ 0.05; ***P* ≤ 0.01; Abbreviations: *APOE* = apolipoprotein E; HR = hazard ratio

SI conversion: To convert cholesterol to millimoles per liter, multiply by 0.0259. Depressive symptoms were scored using the Maastricht Vital Exhaustion Questionnaire and dichotomized at ≥ 14 to indicate depressive symptomology. Effect modification of depressive symptoms on the association between mid-life social relationships and dementia risk was not significant (model 2 *p*-interaction = 0.80). Total N reflects data imputed for 193 participants.

^a^Model adjusted for age, sex, race-center, *APOE*ε4, and educational level.

^b^Model adjusted for model 1 covariates occupational/marital status at visit 2

^c^Model adjusted for model 2 covariates and vascular risk factors measured at visit 2.

**eTable 6. Adjusted Hazard Ratios for Association of Mid-Life Social Relationships with Dementia Risk, Stratified by Education Level (n = 13070)**

|  | **Below High School (n = 2687)** | | | **High School or Equivalent (n = 4107)** | | | **More than High School (n = 6276)** | | |
| --- | --- | --- | --- | --- | --- | --- | --- | --- | --- |
| **Social Relationships** | **Model 1^a^ HR**  **(95% CI)** | **Model 2^b^ HR**  **(95% CI)** | **Model 3^c^ HR**  **(95% CI)** | **Model 1^a^ HR**  **(95% CI)** | **Model 2^b^ HR**  **(95% CI)** | **Model 3^c^ HR**  **(95% CI)** | **Model 1^a^ HR**  **(95% CI)** | **Model 2^b^ HR**  **(95% CI)** | **Model 3^c^ HR**  **(95% CI)** |
| Strong | 0.68  (0.56 – 0.83)** | 0.74  (0.60 – 0.91)** | 0.73  (0.59 – 0.90)** | 0.71  (0.58 – 0.87)** | 0.77  (0.62 – 0.94)* | 0.76  (0.62 – 0.93)** | 0.74  (0.63 - 0.89)** | 0.80  (0.66 – 0.96)* | 0.78  (0.65 – 0.94)** |
| Average | 0.87  (0.71 – 1.08) | 0.92  (0.74 – 1.14) | 0.92  (0.74 – 1.14) | 0.76  (0.61 – 0.95)* | 0.79  (0.63 – 0.98)* | 0.77  (0.62 – 0.96)* | 0.81  (0.67 – 0.98)* | 0.84  (0.69 – 1.02) | 0.82  (0.68 – 1.00) |
| Poor | Reference | Reference | Reference | Reference | Reference | Reference | Reference | Reference | Reference |
| **Age**  **(per 1 year)** | 1.12  (1.11– 1.14)** | 1.12  (1.10 – 1.14)** | 1.12  (1.10 – 1.14)** | 1.16  (1.15 – 1.18)** | 1.15  (1.14 – 1.17)** | 1.15  (1.13 – 1.17)** | 1.18  (1.17 – 1.19)** | 1.18  (1.16 – 1.19)** | 1.18  (1.16 – 1.19)** |
| **Race-ARIC Center** |  |  |  |  |  |  |  |  |  |
| White - Minnesota | 1.01  (0.71 – 1.42) | 1.05  (0.74 – 1.49) | 1.02  (0.71 – 1.45) | 1.07  (0.88 – 1.30) | 1.14  (0.94 – 1.39) | 1.15  (0.93 – 1.42) | 1.11  (0.95 – 1.31) | 1.13  (0.96 – 1.33) | 1.11  (0.94 – 1.31) |
| White – Maryland | 0.93  (0.73 – 1.19) | 0.96  (0.75 – 1.22) | 0.94  (0.74 – 1.20) | 1.08  (0.89 – 1.30) | 1.09  (0.90 – 1.32) | 1.06  (0.87 – 1.29) | 1.26  (1.06 – 1.50)** | 1.26  (1.05 – 1.50)* | 1.22  (1.02 – 1.45)* |
| Black –  Forsyth | 1.46  (0.88 – 2.41) | 1.44  (0.87 – 2.39) | 1.29  (0.77 – 2.15) | 1.28  (0.78 - 2.12) | 1.20  (0.72 – 1.99) | 0.97  (0.58 – 1.62) | 1.19  (0.77 – 1.85) | 1.15  (0.74 – 1.78) | 1.06  (0.68 – 1.65) |
| Black - Jackson | 1.54  (1.22 – 1.93)** | 1.50  (1.19 – 1.89)** | 1.36  (1.07 – 1.73)* | 1.79  (1.43 – 2.25)** | 1.77  (1.40 – 2.24)** | 1.45  (1.13 – 1.86)** | 1.60  (1.33 – 1.93)** | 1.57  (1.31 – 1.89)** | 1.40  (1.15 – 1.70)** |
| White - Forsyth | Reference | Reference | Reference | Reference | Reference | Reference | Reference | Reference | Reference |
| **Sex (Men)** | 0.97  (0.83 – 1.13) | 1.07  (0.91 – 1.26) | 1.06  (0.89 – 1.27) | 1.18  (1.02 – 1.36)* | 1.33  (1.14 – 1.55)** | 1.29  (1.09 – 1.52)** | 1.08  (0.95 – 1.21) | 1.13  (0.99 – 1.29) | 1.12  (0.98 – 1.29) |
| ***APOE***ε**4 Carrier** | 1.58  (1.36 – 1.85)** | 1.58  (1.35 – 1.84)** | 1.59  (1.37 – 1.86)** | 1.82  (1.58 – 2.11)** | 1.85  (1.60 – 2.13)** | 1.87  (1.62 – 2.16)** | 2.18  (1.93 – 2.47)** | 2.19  (1.94 – 2.48)** | 2.19  (1.94 – 2.48)** |
| **Not working outside the home** | ----- | 1.09  (0.93 – 1.27) | 1.06  (0.91 – 1.24) | ----- | 1.28  (1.10 – 1.50)** | 1.27  (1.09 – 1.49)** | ----- | 1.09  (0.95 – 1.25) | 1.08  (0.94 – 1.24) |
| **Not Married** | ----- | 1.20  (1.01 – 1.43)* | 1.16  (0.97 – 1.38) | ----- | 1.24  (1.03 – 1.50)* | 1.25  (1.03 – 1.50)* | ----- | 1.10  (0.93 – 1.30) | 1.09  (0.92 – 1.29) |
| **Depressive Symptoms** | ----- | 1.20  (1.02 – 1.41)* | 1.17  (1.00 – 1.38) | ----- | 1.27  (1.09 – 1.47)** | 1.23  (1.06 – 1.43)** | ----- | 1.16  (0.99 – 1.35) | 1.12  (0.96 – 1.31) |
| **Hypertension** | ----- | ----- | 1.18  (1.01 – 1.38)* | ----- | ----- | 1.18  (1.02 – 1.38)* | ----- | ----- | 1.08  (0.94 – 1.23) |
| **Diabetes** | ----- | ----- | 1.35  (1.12 – 1.63)** | ----- | ----- | 1.95  (1.61 – 2.37)** | ----- | ----- | 1.45  (1.20 – 1.75)** |
| **Body Mass Index**  **(≥ 30 kg/m^2^)** | ----- | ----- | 1.06  (0.90 – 1.25) | ----- | ----- | 1.06  (0.90– 1.25) | ----- | ----- | 1.18  (1.02 – 1.36)* |
| **Total Cholesterol**  **(≥ 200 mg/dL)** | ----- | ----- | 0.93  (0.80 – 1.08) | ----- | ----- | 1.07  (0.93 – 1.24) | ----- | ----- | 1.07  (0.94 – 1.21) |
| **Ever smoker** | ----- | ----- | 1.04  (0.88 – 1.23) | ----- | ----- | 1.21  (1.04 – 1.41)* | ----- | ----- | 1.08  (0.95 – 1.23) |
| **Ever drinker** | ----- | ----- | 0.99  (0.83 –1.18) | ----- | ----- | 0.84  (0.70 – 1.00) | ----- | ----- | 0.92  (0.78 – 1.08) |

**P* ≤ 0.05; ***P* ≤ 0.01; Abbreviations: *APOE* = apolipoprotein E; HR = hazard ratio. SI conversion: To convert cholesterol to millimoles per liter, multiply by 0.0259. Effect modification of education level on the association between social relationships and dementia risk was not significant (*p*-interaction = 0.39). Total N reflects data imputed for 18 participants.

^a^Model adjusted for age, sex, race-center, and *APOE*ε4

^b^Model adjusted for model 1 covariates, occupational/marital status and (having) depressive symptoms at visit 2

^c^Model adjusted for model 2 covariates and vascular risk factors measured at visit 2.

**eTable 7. Adjusted Hazard Ratios for Association of Mid-Life Social Support with Dementia Risk (n = 13070)**

| **Social Support** | **Model 1^a^ HR**  **(95% CI)** | **Model 2^b^ HR**  **(95% CI)** | **Model 3^c^ HR**  **(95% CI)** |
| --- | --- | --- | --- |
| High (n = 3961) | 0.79 (0.72 – 0.87)** | 0.84 (0.76 – 0.93)** | 0.83 (0.76 – 0.92)** |
| Intermediate (n = 4555) | 0.80 (0.73 - 0.88)** | 0.83 (0.76 – 0.91)** | 0.83 (0.76 – 0.91)** |
| Low (n = 4824) | Reference | Reference | Reference |
| **Age (per 1 year)** | 1.16 (1.15 - 1.17)** | 1.15 (1.14 - 1.16)** | 1.15 (1.14 - 1.16)** |
| **Race-ARIC Center** |  |  |  |
| White - Minnesota | 1.07 (0.95 – 1.20) | 1.11 (0.99 – 1.24) | 1.09 (0.97 – 1.23) |
| White – Maryland | 1.11 (0.99 – 1.24) | 1.12 (1.00 – 1.26)* | 1.09 (0.97 – 1.22) |
| Black – Forsyth | 1.31 (0.99 - 1.72) | 1.24 (0.94 – 1.63) | 1.10 (0.84 – 1.45) |
| Black - Jackson | 1.66 (1.47 – 1.87)** | 1.60 (1.42 – 1.81)** | 1.41 (1.24 – 1.59)** |
| White - Forsyth | Reference | Reference | Reference |
| **Sex (Men)** | 1.06 (0.98 – 1.15) | 1.17 (1.07 – 1.27)** | 1.15 (1.05 – 1.26)** |
| ***APOE***ε**4 Carrier** | 1.89 (1.74 – 2.04)** | 1.89 (1.75 – 2.05)** | 1.91 (1.76 – 2.06)** |
| **Education Level** |  |  |  |
| Less than high school | Reference | Reference | Reference |
| High school or equivalent | 0.76 (0.68 – 0.84)** | 0.78 (0.70 – 0.87)** | 0.80 (0.72 – 0.89)** |
| More than high school | 0.64 (0.58 – 0.71)** | 0.68 (0.61 – 0.75)** | 0.70 (0.63 – 0.77)** |
| **Not working outside the home** | ----- | 1.15 (1.06 – 1.26)** | 1.13 (1.04 – 1.23)** |
| **Not Married** | ----- | 1.21 (1.10 – 1.34)** | 1.19 (1.07 – 1.31)** |
| **Depressive Symptoms** | ----- | 1.21 (1.10 – 1.32)** | 1.17 (1.07 – 1.29)** |
| **Hypertension** | ----- | ----- | 1.16 (1.07 – 1.26)** |
| **Diabetes** | ----- | ----- | 1.52 (1.37 - 1.70)** |
| **Body Mass Index (≥ 30 kg/m^2^)** | ----- | ----- | 1.12 (1.02 – 1.22)* |
| **Total Cholesterol (≥ 200 mg/dL)** | ----- | ----- | 1.03 (0.96 – 1.12) |
| **Ever smoker** | ----- | ----- | 1.12 (1.03 – 1.22)** |
| **Ever drinker** | ----- | ----- | 0.92 (0.84 – 1.02) |

**P* ≤ 0.05; ***P* ≤ 0.01; Abbreviations: *APOE* = apolipoprotein E; HR = hazard ratio. SI conversion: To convert cholesterol to millimoles per liter, multiply by 0.0259.

^a^ Model adjusted for age, race, sex, *APOE*ε4, education

^b^ Model adjusted for model 1 covariates, occupational/marital status and (having) depressive symptoms as measured at visit 2

^c^ Model adjusted for model 2 covariates and vascular risk factors as measured at visit 2

**eTable 8. Adjusted Hazard Ratios for Association of Mid-Life Social Isolation with Dementia Risk (n = 13070)**

| **Social Isolation** | **Model 1^a^ HR**  **(95% CI)** | **Model 2^b^ HR**  **(95% CI)** | **Model 3^c^ HR**  **(95% CI)** |
| --- | --- | --- | --- |
| Low risk (n = 10204) | 0.64 (0.51 – 0.79)** | 0.71 (0.57 – 0.88)** | 0.70 (0.56 – 0.87)** |
| Moderate risk (n = 1776) | 0.68 (0.53 - 0.86)** | 0.72 (0.57 – 0.91)** | 0.73 (0.57 – 0.92)** |
| High risk (n = 731) | 0.91 (0.70 – 1.19) | 0.93 (0.72 – 1.21) | 0.94 (0.72 – 1.22) |
| Isolated (n =359) | Reference | Reference | Reference |
| **Age (per 1 year)** | 1.16 (1.15 - 1.17)** | 1.15 (1.14 - 1.16)** | 1.15 (1.14 - 1.16)** |
| **Race-ARIC Center** |  |  |  |
| White - Minnesota | 1.08 (0.96 – 1.21) | 1.12 (1.00 – 1.26) | 1.10 (0.98 – 1.24) |
| White – Maryland | 1.10 (0.98 – 1.24) | 1.12 (1.00 – 1.25) | 1.09 (0.97 – 1.22) |
| Black – Forsyth | 1.32 (1.00 – 1.74)* | 1.26 (0.96 – 1.66) | 1.11 (0.84 – 1.47) |
| Black - Jackson | 1.65 (1.46 – 1.85)** | 1.61 (1.43 – 1.81)** | 1.41 (1.24 – 1.60)** |
| White - Forsyth | Reference | Reference | Reference |
| **Sex (Men)** | 1.06 (0.98 – 1.15) | 1.16 (1.07 – 1.27)** | 1.15 (1.05 – 1.26)** |
| ***APOE***ε**4 Carrier** | 1.88 (1.74 – 2.04)** | 1.89 (1.75 – 2.05)** | 1.90 (1.75 – 2.06)** |
| **Education Level** |  |  |  |
| Less than high school | Reference | Reference | Reference |
| High school or equivalent | 0.76 (0.68 – 0.84)** | 0.78 (0.70 – 0.87)** | 0.80 (0.72 – 0.89)** |
| More than high school | 0.64 (0.57 – 0.70)** | 0.67 (0.60 – 0.74)** | 0.69 (0.63 – 0.77)** |
| **Not working outside the home** | ----- | 1.15 (1.05 – 1.25)** | 1.12 (1.03 – 1.23)** |
| **Not Married** | ----- | 1.16 (1.05 – 1.29)** | 1.13 (1.02 – 1.26)* |
| **Depressive Symptoms** | ----- | 1.24 (1.14 – 1.36)** | 1.21 (1.11 – 1.32)** |
| **Hypertension** | ----- | ----- | 1.16 (1.07 – 1.26)** |
| **Diabetes** | ----- | ----- | 1.52 (1.36 - 1.70)** |
| **Body Mass Index (≥ 30 kg/m^2^)** | ----- | ----- | 1.12 (1.03 – 1.23)* |
| **Total Cholesterol (≥ 200 mg/dL)** | ----- | ----- | 1.03 (0.95 – 1.12) |
| **Ever smoker** | ----- | ----- | 1.12 (1.03 – 1.22)** |
| **Ever drinker** | ----- | ----- | 0.92 (0.83 – 1.01) |

**P* ≤ 0.05; ***P* ≤ 0.01; Abbreviations: *APOE* = apolipoprotein E; HR = hazard ratio. SI conversion: To convert cholesterol to millimoles per liter, multiply by 0.0259.

^a^ Model adjusted for age, race, sex, *APOE*ε4, education

^b^ Model adjusted for model 1 covariates, occupational/marital status and (having) depressive symptoms as measured at visit 2.

^c^ Model adjusted for model 2 covariates and vascular risk factors as measured at visit 2.

**eTable 9. Adjusted Hazard Ratios for Association of Mid-Life Social Relationships with Dementia Risk, Excluding those with Global Cognition in Lowest 5^th^ Percentile at Visit 2 (n = 12448)**

| **Social Relationships** | **Model 1^a^ HR**  **(95% CI)** | **Model 2^b^ HR**  **(95% CI)** | **Model 3^c^ HR**  **(95% CI)** |
| --- | --- | --- | --- |
| Strong | 0.71 (0.64 – 0.80)** | 0.77 (0.68 – 0.87)** | 0.75 (0.67 – 0.85)** |
| Average | 0.81 (0.72 – 0.92)** | 0.84 (0.74 – 0.96)** | 0.83 (0.73 – 0.95)** |
| Poor | Reference | Reference | Reference |
| **Age (per 1 year)** | 1.16 (1.15 - 1.17)** | 1.16 (1.15 - 1.17)** | 1.15 (1.14 - 1.16)** |
| **Race-ARIC Center** |  |  |  |
| White - Minnesota | 1.08 (0.96 – 1.22) | 1.12 (0.99 – 1.26) | 1.10 (0.97 – 1.24) |
| White – Maryland | 1.12 (1.00 – 1.26)* | 1.13 (1.01 – 1.27)* | 1.10 (0.98 – 1.23) |
| Black – Forsyth | 1.30 (0.98 - 1.72) | 1.25 (0.94 – 1.65) | 1.10 (0.83 – 1.46) |
| Black - Jackson | 1.53 (1.35 – 1.73)** | 1.49 (1.31 – 1.69)** | 1.29 (1.13 – 1.47)** |
| White - Forsyth | Reference | Reference | Reference |
| **Sex (Men)** | 1.04 (0.95 – 1.13) | 1.13 (1.03 – 1.23)** | 1.11 (1.01 – 1.22)* |
| ***APOE***ε**4 Carrier** | 1.96 (1.81 – 2.13)** | 1.97 (1.82 – 2.14)** | 1.99 (1.83 – 2.16)** |
| **Education Level** |  |  |  |
| Less than high school | Reference | Reference | Reference |
| High school or equivalent | 0.79 (0.70 – 0.88)** | 0.80 (0.72 – 0.90)** | 0.82 (0.73 – 0.92)** |
| More than high school | 0.67 (0.61 – 0.75)** | 0.70 (0.63 – 0.78)** | 0.73 (0.65 – 0.81)** |
| **Not working outside the home** | ----- | 1.15 (1.05 – 1.26)** | 1.13 (1.03 – 1.24)** |
| **Not Married** | ----- | 1.15 (1.04 – 1.28)** | 1.13 (1.02 – 1.26)* |
| **Depressive Symptoms** | ----- | 1.20 (1.10 – 1.32)** | 1.17 (1.07 – 1.28)** |
| **Hypertension** | ----- | ----- | 1.15 (1.05 – 1.26)** |
| **Diabetes** | ----- | ----- | 1.55 (1.38 - 1.74)** |
| **Body Mass Index (≥ 30 kg/m^2^)** | ----- | ----- | 1.17 (1.06 – 1.28)** |
| **Total Cholesterol (≥ 200 mg/dL)** | ----- | ----- | 1.02 (0.94 – 1.11) |
| **Ever smoker** | ----- | ----- | 1.12 (1.03 – 1.23)* |
| **Ever drinker** | ----- | ----- | 0.92 (0.83 – 1.02) |

**P* ≤ 0.05; ***P* ≤ 0.01; Abbreviations: *APOE* = apolipoprotein E; HR = hazard ratio. SI conversion: To convert cholesterol to millimoles per liter, multiply by 0.0259.

^a^ Model adjusted for age, race, sex, *APOE*ε4, education

^b^ Model adjusted for model 1 covariates, occupational/marital status and (having) depressive symptoms as measured at visit 2.

^c^ Model adjusted for model 2 covariates and vascular risk factors as measured at visit 2.

**eTable 10. Adjusted Hazard Ratios for Association of Mid-Life Social Relationships with Dementia Risk, Excluding those who had Incident Stroke before End of Follow-up (n = 11708)**

| **Social Relationships** | **Model 1^a^ HR**  **(95% CI)** | **Model 2^b^ HR**  **(95% CI)** | **Model 3^c^ HR**  **(95% CI)** |
| --- | --- | --- | --- |
| Strong | 0.69 (0.62 – 0.78)** | 0.75 (0.66 – 0.85)** | 0.74 (0.65 – 0.83)** |
| Average | 0.81 (0.71 – 0.92)** | 0.84 (0.73 – 0.96)** | 0.83 (0.73 – 0.95)** |
| Poor | Reference | Reference | Reference |
| **Age (per 1 year)** | 1.17 (1.16 - 1.18)** | 1.16 (1.15 - 1.17)** | 1.16 (1.15 - 1.17)** |
| **Race-ARIC Center** |  |  |  |
| White - Minnesota | 1.07 (0.95 – 1.22) | 1.11 (0.98 – 1.26)* | 1.09 (0.96 – 1.25) |
| White – Maryland | 1.07 (0.95 – 1.22) | 1.08 (0.96 – 1.23) | 1.06 (0.93 – 1.20) |
| Black – Forsyth | 1.14 (0.83 - 1.57) | 1.09 (0.79 – 1.50) | 0.99 (0.72 – 1.37) |
| Black - Jackson | 1.56 (1.37 – 1.78)** | 1.52 (1.33 – 1.74)** | 1.35 (1.17 – 1.55)** |
| White - Forsyth | Reference | Reference | Reference |
| **Sex (Men)** | 1.03 (0.94 – 1.12) | 1.12 (1.02 – 1.23)* | 1.11 (1.00 – 1.22)* |
| ***APOE***ε**4 Carrier** | 1.94 (1.77 – 2.11)** | 1.94 (1.78 – 2.12)** | 1.95 (1.79 – 2.13)** |
| **Education Level** |  |  |  |
| Less than high school | Reference | Reference | Reference |
| High school or equivalent | 0.74 (0.66 – 0.83)** | 0.76 (0.67 – 0.85)** | 0.77 (0.69 – 0.87)** |
| More than high school | 0.64 (0.57 – 0.72)** | 0.67 (0.60 – 0.75)** | 0.69 (0.62 – 0.77)** |
| **Not working outside the home** | ----- | 1.14 (1.04 – 1.26)** | 1.13 (1.02 – 1.24)* |
| **Not Married** | ----- | 1.16 (1.04 – 1.29)** | 1.13 (1.01 – 1.26)* |
| **Depressive Symptoms** | ----- | 1.21 (1.09 – 1.33)** | 1.18 (1.07 – 1.30)** |
| **Hypertension** | ----- | ----- | 1.12 (1.02 – 1.23)* |
| **Diabetes** | ----- | ----- | 1.49 (1.32 - 1.69)** |
| **Body Mass Index (≥ 30 kg/m^2^)** | ----- | ----- | 1.14 (1.04 – 1.26)** |
| **Total Cholesterol (≥ 200 mg/dL)** | ----- | ----- | 1.05 (0.96 – 1.14) |
| **Ever smoker** | ----- | ----- | 1.12 (1.02 – 1.23)* |
| **Ever drinker** | ----- | ----- | 0.92 (0.82 – 1.02) |

**P* ≤ 0.05; ***P* ≤ 0.01; Abbreviations: *APOE* = apolipoprotein E; HR = hazard ratio. SI conversion: To convert cholesterol to millimoles per liter, multiply by 0.0259. Participants who had a stroke *after* developing dementia were part of this exclusion.

^a^ Model adjusted for age, race, sex, *APOE*ε4, education

^b^ Model adjusted for model 1 covariates, occupational/marital status and (having) depressive symptoms as measured at visit 2.

^c^ Model adjusted for model 2 covariates and vascular risk factors as measured at visit 2.

**eTable 11. Adjusted Sub-Hazard Ratios for Association of Mid-Life Relationships with Dementia Risk, Accounting for Competing Risk of Death (n = 13070)**

| **Social Relationships** | **Model 1^a^ HR**  **(95% CI)** | **Model 2^b^ HR**  **(95% CI)** | **Model 3^c^ HR**  **(95% CI)** |
| --- | --- | --- | --- |
| Strong | 0.84 (0.75 – 0.94)** | 0.86 (0.76 – 0.96)** | 0.85 (0.75 – 0.95)** |
| Average | 0.88 (0.78 – 0.99)* | 0.88 (0.78 – 1.00)* | 0.88 (0.77 – 0.99)* |
| Poor | Reference | Reference | Reference |
| **Age (per 1 year)** | 1.10 (1.10 – 1.11)** | 1.10 (1.10 - 1.11)** | 1.10 (1.09 - 1.11)** |
| **Race-ARIC Center** |  |  |  |
| White - Minnesota | 1.11 (0.99 – 1.25) | 1.11 (0.99 – 1.25) | 1.14 (1.01 – 1.28)* |
| White – Maryland | 1.18 (1.05 – 1.32)** | 1.18 (1.05 – 1.32)** | 1.17 (1.04 – 1.31)** |
| Black – Forsyth | 1.04 (0.78 – 1.38) | 1.03 (0.77 - 1.37) | 1.01 (0.75 – 1.35) |
| Black - Jackson | 1.47 (1.30 – 1.65)** | 1.46 (1.29 – 1.65)** | 1.40 (1.23 – 1.59)** |
| White - Forsyth | Reference | Reference | Reference |
| **Sex (Men)** | 0.82 (0.76 – 0.89)** | 0.83 (0.76 – 0.91)** | 0.89 (0.81 – 0.97)* |
| ***APOE***ε**4 Carrier** | 1.79 (1.65 – 1.94)** | 1.79 (1.65 – 1.94)** | 1.79 (1.65 – 1.95)** |
| **Education Level** |  |  |  |
| Less than high school | Reference | Reference | Reference |
| High school or equivalent | 0.90 (0.81 – 1.00) | 0.91 (0.81 – 1.01) | 0.90 (0.81 – 1.01) |
| More than high school | 0.80 (0.72 – 0.89)** | 0.81 (0.73 – 0.90)** | 0.81 (0.73 – 0.90)** |
| **Not working outside the home** | ----- | 1.00 (0.91 – 1.09) | 1.00 (0.91 – 1.09) |
| **Not Married** | ----- | 1.02 (0.92 – 1.12) | 1.03 (0.93 – 1.14) |
| **Depressive Symptoms** | ----- | 1.05 (0.96 – 1.15) | 1.04 (0.95 – 1.14) |
| **Hypertension** | ----- | ----- | 1.02 (0.94 – 1.11) |
| **Diabetes** | ----- | ----- | 1.05 (0.94 – 1.18) |
| **Body Mass Index (≥ 30 kg/m^2^)** | ----- | ----- | 1.03 (0.94 – 1.13) |
| **Total Cholesterol (≥ 200 mg/dL)** | ----- | ----- | 1.05 (0.97 – 1.14) |
| **Ever smoker** | ----- | ----- | 0.87 (0.80 – 0.95)** |
| **Ever drinker** | ----- | ----- | 0.91 (0.82 – 1.00) |

**P* ≤ 0.05; ***P* ≤ 0.01; Abbreviations: *APOE* = apolipoprotein E; HR = hazard ratio. SI conversion: To convert cholesterol to millimoles per liter, multiply by 0.0259.

^a^ Model adjusted for age, race, sex, *APOE*ε4, education

^b^ Model adjusted for model 1 covariates, occupational/marital status and (having) depressive symptoms as measured at visit 2.

^c^ Model adjusted for model 2 covariates and vascular risk factors as measured at visit 2.
